# Synthesis and Structures of Lead(II) Complexes with Substituted Derivatives of the *Closo*-Decaborate Anion with a Pendant N_3_ Group

**DOI:** 10.3390/molecules28248073

**Published:** 2023-12-13

**Authors:** Evgenii Yu. Matveev, Olga S. Dontsova, Varvara V. Avdeeva, Alexey S. Kubasov, Andrey P. Zhdanov, Svetlana E. Nikiforova, Lyudmila V. Goeva, Konstantin Yu. Zhizhin, Elena A. Malinina, Nikolay T. Kuznetsov

**Affiliations:** 1 Institute of Fine Chemical Technologies Named after M. V. Lomonosov, MIREA—Russian Technological University, Vernadskogo pr. 86, Moscow 119571, Russia; cat1983@yandex.com (E.Y.M.); olgadoncovasp@gmail.com (O.S.D.); zhizhin@igic.ras.ru (K.Y.Z.); 2 Kurnakov Institute of General and Inorganic Chemistry, Russian Academy of Sciences, Leninskii pr. 31, Moscow 119991, Russia; fobosax@mail.ru (A.S.K.); zhdanov@igic.ras.ru (A.P.Z.); korolencko0110@yandex.ru (S.E.N.); lyudmila.goeva@mail.ru (L.V.G.); malinina@igic.ras.ru (E.A.M.); ntkuz@igic.ras.ru (N.T.K.)

**Keywords:** boron cluster anions, decahydro-*closo*-decaborate anion, decahydrido-*closo*-decaborate anion, complexes, lead, non-covalent interactions

## Abstract

In this work, we studied lead(II) and cobalt(II) complexation of derivatives [2-B_10_H_9_O(CH_2_)_2_O(CH_2_)_2_N_3_]^2−^ and [2-B_10_H_9_O(CH_2_)_5_N_3_]^2−^ of the *closo*-decaborate anion containing pendant azido groups in the presence of 1,10-phenanthroline and 2,2′-bipyridyl. Mononuclear [PbL_2_{An}] and binuclear [Pb_2_L_4_(NO_3_)_2_{An}] lead complexes (where {An} is the N_3_-substituted boron cluster) were isolated and studied by IR spectroscopy and elemental analysis. The mononuclear lead(II) complex [Pb(phen)_2_[B_10_H_9_O(CH_2_)_2_O(CH_2_)_2_N_3_] and the binuclear lead(II) complex [Pb_2_(phen)_4_(NO_3_)_2_[B_10_H_9_O(CH_2_)_5_)N_3_] were determined by single-crystal X-ray diffraction. In complex [Pb_2_(phen)_4_(NO_3_)_2_[B_10_H_9_O(CH_2_)_5_)N_3_], the boron cluster is coordinated by the metal atom only via the 3c2e MHB bonds. In complex [Pb(phen)_2_[B_10_H_9_O(CH_2_)_2_O(CH_2_)_2_N_3_], the coordination environment of the metal includes BH groups of the boron cluster and the oxygen atom of the exo-polyhedral substituent. When the reaction was performed in a CH_3_CN/water mixture, the binuclear lead(II) complex [(Pb(bipy)NO_3_)(Pb(bipy)_2_NO_3_)(B_10_H_9_O(CH_2_)_2_O(CH_2_)_2_N_3_)]·CH_3_CN·H_2_O was isolated, where the boron cluster acts as a bridging ligand between lead atoms coordinated by the boron cage via the O atoms of the substituent and/or the BH groups. In the course of cobalt(II) complexation, the starting compound (Ph_4_P)_2_[B_10_H_9_O(CH_2_)_5_N_3_] was isolated and its structure was also determined by X-ray diffraction. Although a number of lead(II) complexes with coordinated N_3_ are known from the literature, no complexes with the boron cluster coordinated by the pendant N_3_ group involved in the metal coordination have been isolated.

## 1. Introduction

Boron cluster anions [B*_n_*H*_n_*]^2−^ (*n* = 6–12) attract attention as completely inorganic clusters with high resistance to elevated temperatures and oxidizing agents, but at the same time are quite easily subjected to direct functionalization due to the substitution of terminal hydrogen atoms with various functional groups [1,2,3,4,5]. The derivatives obtained can be used in further modification reactions, for example, due to the presence of thiol [6,7,8,9], nitrile [10,11,12], hydroxyl [13,14,15], and other groups. Compounds based on the most well-known *closo*-borate anions, [B_10_H_10_]^2−^ and [B_12_H_12_]^2−^, are actively studied and used in various fields of science and technology, for example, in the creation of energy storage systems, the synthesis of composite materials, materials for electronics, etc. [16,17,18,19,20]. Preparations based on boron cluster anions and carboranes are used for diagnostics and neutron capture therapy of malignant tumors (^10^B NCT) [21,22,23] and have antimicrobial [24,25] and antiviral [26,27,28] effects. In this regard, the search for new methods for the functionalization of [B_10_H_10_]^2−^ and [B_12_H_12_]^2−^ anions is an urgent task.

Derivatives of higher boron anions [B*_n_*H*_n_*]^2−^ (*n* = 10, 12) with cyclic substituents of the oxonium type are easily modified, affording *closo*-borates with pendant functional groups [29,30,31,32,33,34,35,36,37]. These reactions proceed under the action of a wide range of nucleophilic reagents and are accompanied by the opening of a cyclic substituent. Functional groups attached through the alkoxy spacer chain and isolated in this way can enter into further functionalization reactions, including fragments of biologically active molecules [32,38,39]. Boron cluster anion derivatives containing a pendant azido group are of particular interest as objects for direct application in click chemistry [40,41,42,43].

Anions [B*_n_*H*_n_*]^2−^ (*n* = 10, 12) and their derivatives are currently of considerable interest in the synthesis of complex compounds. To date, quite a lot of examples of their application both as counterions [44,45,46] and as polydentate and polytope ligands [47,48] have been described. *Closo*-borates with pendant functional groups have great potential in complexation due to the presence of several coordination centers: a Pearson soft boron cluster, an alkoxy spacer group, and an introduced pendant fragment. Varying the structure of the spacer (both by length and the presence of donor atoms of various types) and the pendant fragment opens up great prospects in the synthesis of metal coordination compounds with different Pearson hardness/softness levels, as well as complexes with the desired structures and properties. For example, the lead(II) complexation with hydroxy-*closo*-decaborates with different types of binding of the OH group to the boron cluster leads to mono-, binuclear, and polymeric complexes [49]. Research in this area is of interest both from the point of view of fundamental science and from the point of view of practice, for example, for the synthesis of functional boron-containing materials [50,51].

It is known that the azide ion is able to be coordinated by lead(II) in various ways; in particular, it can act as a tridentate (μ_1,1,1_-N_3_ or μ_1,1,3_-N_3_) and a bidentate bridging ligand (μ_1,1_-N_3_ or μ_1,3_-N_3_) [52]. The obtained derivatives can be used in the synthesis of organometallic coordination polymers; therefore, studies in this field of lead(II) coordination chemistry are topical.

The aim of this work is to synthesize and study the *closo*-decaborate anion derivatives containing pendant azido groups in lead(II) and cobalt(II) complexation in the presence of azaheterocyclic ligands, namely 1,10-phenanthroline and 2,2′-bipyridyl.

## 2. Results and Discussion

**Scheme 1:**
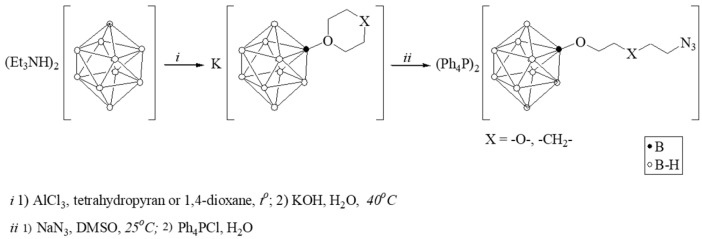
Synthesis of substituted derivatives [2-B_10_H_9_RN_3_]^2−^, where R = O(CH_2_)_2_O(CH_2_)_2_) (**1**) or O(CH_2_)_5_ (**2**).

Derivatives of the *closo*-decaborate anion containing a pendant N_3_ group [2-B_10_H_9_RN_3_]^2−^ (where R = O(CH_2_)_2_O(CH_2_)_2_) (**1**) or O(CH_2_)_5_ (**2**)) were obtained by multistage synthesis starting from the [B_10_H_10_]^2−^ anion (Scheme 1), as reported in [53].

Compounds **1** and **2** were synthesized according to the known procedure reported in [53]. The introduction of an *exo*-polyhedral substituent and its subsequent opening with the formation of *closo*-decaborates containing pendant azido groups were monitored using ^11^B and ^11^B{^1^H} NMR spectroscopy. For example, in the ^11^B{^1^H} NMR spectrum of the *closo*-decaborate anion, there were only two signals with an integral intensity ratio of 1:4 from two apical and eight equatorial boron atoms, respectively. In the ^11^B{^1^H} spectrum of the product of the reaction with 1,4-dioxane and salt K[B_10_H_9_O(CH_2_)_4_O], there was one signal at 7.7 ppm from the *ipso*-boron atom, two signals at 0.8 and 6.4 ppm assigned to two chemically non-equivalent apical vertices, two signals at −21.5 and −23.4 ppm corresponding to two pairs of equatorial boron atoms adjacent to the *ipso* position, and one signal at −29.9 ppm assigned to three boron atoms opposite the substituted position. Only one signal at 7.7 ppm did not split into a doublet in the ^11^B NMR spectrum. This spectral pattern makes it possible to unambiguously determine the type of compounds obtained. The ^11^B{^1^H} NMR spectrum of the product of the reaction of the 1,4-dioxane derivative with the [B_10_H_10_]^2−^ anion with sodium azide retained the general form characteristic of *closo*-decaborates with a substituent in the equatorial belt; however, it exhibited a significant rearrangement of signals from boron atoms when compared with the spectrum of the initial derivative of the *closo*-decaborate anion. In particular, the signal from the ipso boron atom at 7.7 ppm shifted towards the strong field up to −1.9 ppm due to a change in the type of the substituent atom from oxonium to alkoxy and a decrease in the polarity of the B–O bond. The signals from two nonequivalent apical vertices of the polyhedron approached each other in the spectrum of the reaction product and changed their positions from −0.8 and −6.4 ppm to −3.8 and −4.8 ppm, respectively. These characteristic changes in the spectra of the products were associated with changes in the valence and charge of the oxonium oxygen atom. The structure of the *exo*-polyhedral substituent was determined by ^1^H NMR and IR spectroscopies.

The obtained compounds **1** and **2** can be considered organic azides with a framework substituent in the form of a boron cluster. It should also be noted that the obtained compounds are very stable in light and air, and in solutions up to a temperature of 100 °C they do not undergo transformations in the azido group. In terms of thermal and kinetic stability, they can be compared with aryl and adamantylazide, which are known coordination compounds with transition metals. In these complexes, the organoazide group shows various coordination possibilities. The organoazide can be monodentate coordinated; this type is characteristic of copper, silver, cobalt, and palladium complexes [54,55,56,57,58]. μ^1,1^-N_3_ bridging coordination is known for a heterometallic zirconium–iridium complex [59]. η^2^-Coordination is observed in the case of some nickel complexes [60]. Quite a large group is formed by complexes with μ^1,1,1^-N_3_ coordination of the azido moiety; this type of binding is characteristic of tantalum, vanadium, tungsten, and titanium ions [61,62,63,64,65,66]. Often, in complexes of this type, the azide moiety is spontaneously or by thermal effect converted to an imido species by extrusion of the N_2_ molecule.

**Scheme 2:**
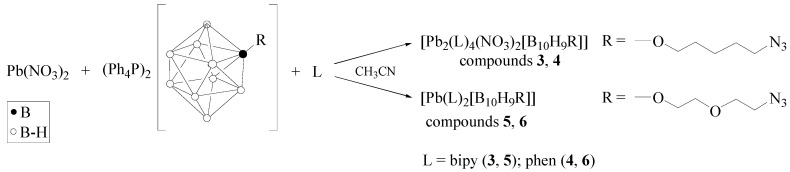
Synthesis of lead(II) complexes **3**–**6** with substituted derivatives [2-B_10_H_9_R]^2−^ in acetonitrile.

Substituted derivatives with a pendant N_3_ group were subsequently used as ligands in lead(II) complexation reactions in the presence of organic ligands phen and bipy. Solid lead(II) nitrate was added to a solution of salt (Ph_4_P)_2_[B_10_H_9_R] in acetonitrile (R = O(CH_2_)_2_O(CH_2_)_2_) or O(CH_2_)_5_). Organic ligands bipy and phen were added to the reaction solution (in a metal-to-ligand ratio of 1:2) to stabilize the lead(II) complexes formed. The complexation was carried out according to Scheme 2.

The final compounds are mononuclear lead(II) complexes [PbL_2_[B_10_H_9_O(CH_2_)_2_O(CH_2_)_2_N_3_] (L = bipy (**3**), phen (**4**)) and binuclear lead(II) complexes [Pb_2_L_4_(NO_3_)_2_[B_10_H_9_O(CH_2_)_5_)N_3_] (L = bipy (**5**), phen (**6**)). The IR spectra of the obtained compounds **3**–**6** contain a broad, intense band of stretching vibrations ν(BH) near 2400 cm^−1^ assigned to uncoordinated BH bonds and a band ν(BH)_MHB_, which is observed as a shoulder in the region of 2300–2200 cm^−1^. The presence of bands in the region 2300–2200 cm^−1^ indicates the coordination of the boron cage via the MHB bond. A broad band of the stretching vibrations ν(NO) is observed in the IR spectra of complexes **5** and **6**. The spectra of all compounds contain bands in the region 1700–600 cm^−1^, which indicate the presence of coordinated bipy and phen molecules.

We succeeded in isolating single crystals of complexes **4** and **6**·3CH_3_CN, which were suitable for further X-ray diffraction studies.

**Figure 1:**
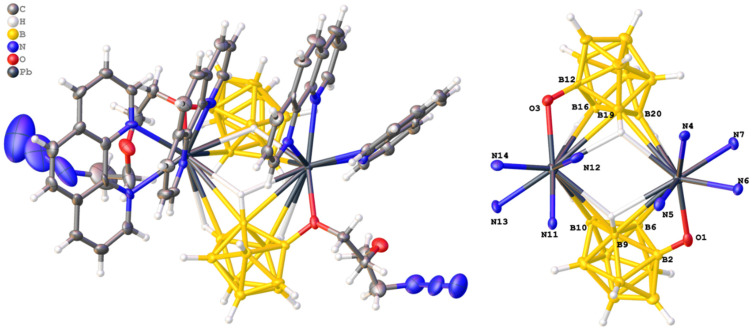
Structure of complex **4** (**left**) and coordination environment of lead atoms in the complex (**right**).

The crystallographically independent part of the triclinic unit cell (P-1) of complex **4** includes the binuclear complex [Pb(phen)_2_[µ-B_10_H_9_O(C_2_H_4_)O(C_2_H_4_)N_3_]]_2_ (Figure 1). Anions [B_10_H_9_O(C_2_H_4_)O(C_2_H_4_)N_3_]^2−^ act as bridging ligands; each anion is coordinated to one lead atom through the oxygen atom O1(O3) and the B6B9 edge (B16B19) and to the second metal atom along the B9B10 edge (B19B20), forming two pairs of three-center two-electron Pb–HBBH bonds. Thus, the coordination environment of each lead atom includes four nitrogen atoms from two phenanthroline molecules, an oxygen atom of the exo-polyhedral substituent, and two edges, B6B9 and B9B10, from two anions (Figure 1). The Pb–O bond lengths are 2.443(7) and 2.562(7) Å, the Pb–B bond lengths fall in the range 3.358(12)–3.593(11) Å, and the Pb–H bond lengths are 3.044(2)–3.7752(14) Å (Table 1). The exo-polyhedral substituent of one of the anions is disordered with a population of 0.65:0.35, with large thermal vibrations of the azide group.

**Figure 2:**
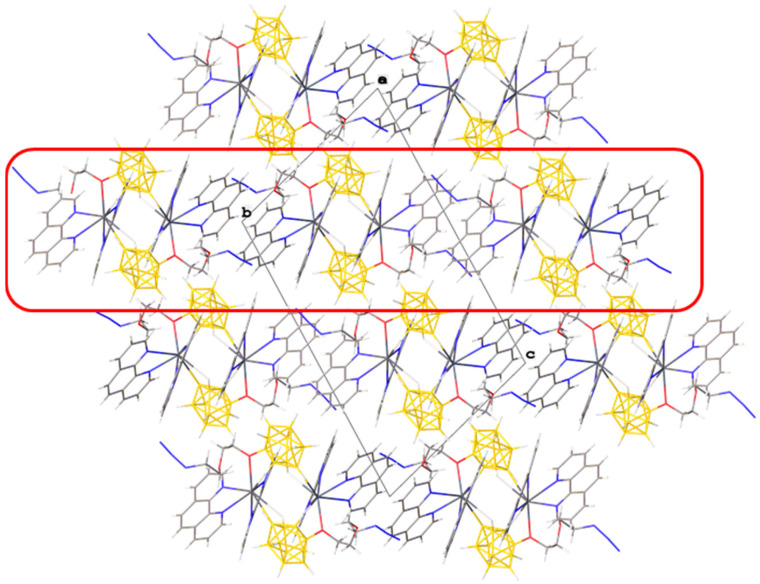
Packing diagram of complex **4**.

Almost all phenanthroline ligands are slightly distorted from a perfectly flat state (RSMD ranges from 0.038 to 0.066 Å). The angles between the PbNN and phen planes lie in the range of 153.9°–167.3°, which is associated with the formation of intra- and intermolecular π–π stacking interactions between the ligands. Molecules connected in this way form 1D polymer chains (Figure 2), which are interconnected through the CH…HB contacts.

**Figure 3:**
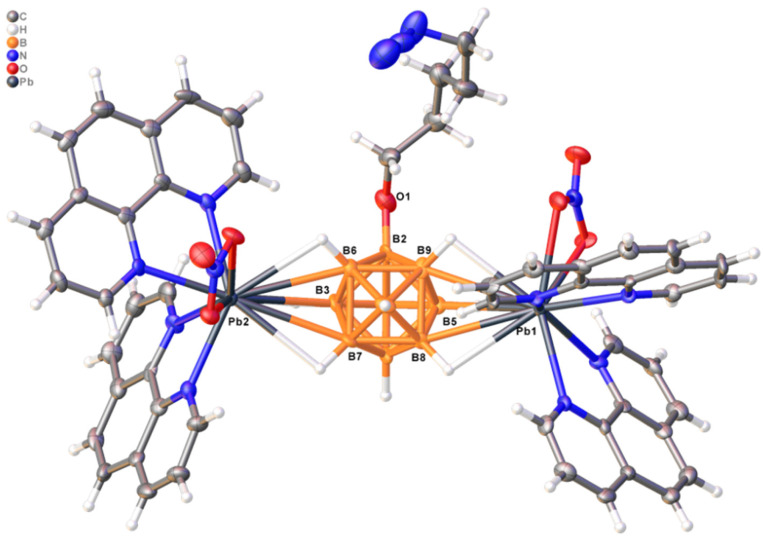
Structure of complex **6**·3CH_3_CN.

**Figure 4:**
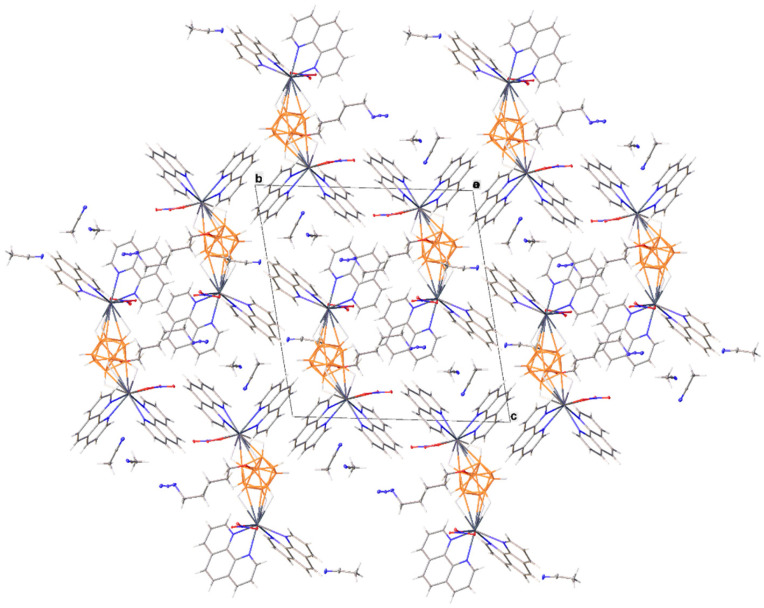
Packing diagram of complex **6**·3CH_3_CN.

The crystallographically independent part of the triclinic unit cell (*P*-1) of complex **6**·3CH_3_CN contains the binuclear complex [(Pb_2_(Phen)_4_(NO_3_))_2_[µ-B_10_H_9_O(CH_2_)_5_N_3_]], in which the [B_10_H_9_O(CH_2_)_5_N_3_]^2−^ anion acts as a bridging ligand, and there are three solvate molecules of acetonitrile (Figure 3). The coordination environment of each lead atom includes two phen molecules, one nitrate ion, and one B5B8B9 and B3B6B7 face forming the environment of the Pb1 and Pb2 atoms, respectively. The Pb–B bond lengths in complex **6**·3CH_3_CN are significantly shorter than those for complex **4**, on average by 0.23 Å (Table 2). The average value of Pb–H contacts in complex **6** is shorter by 0.5 Å. The exo-polyhedral substituent in this case does not participate in coordination and is bonded to neighboring complexes only through CH…O and CH…N contacts. The angles between the PbNN and phen planes lie in the range of 163.8°–178.4°, which is also associated with the formation of π–π stacking interactions between phenanthroline ligands. Solvent molecules are located in channels of square cross-sections parallel to axis a (Figure 4).

**Scheme 3:**
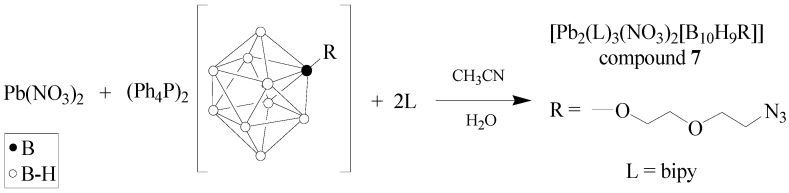
Synthesis of lead(II) complex 7 with substituted derivative [2-B_10_H_9_R]^2−^ (R = O(CH_2_)_2_O(CH_2_)_2_)N_3_) in acetonitrile.

It is interesting that when we used the water/acetonitrile mixture to perform the lead complexation reaction (instead of CH_3_CN, used to prepare complexes **3**–**6**), we succeeded in isolating complex **7** for starting compound **1** (see Scheme 3).

In other cases, no boron-containing complexes were isolated; for example, crystals [Pb(bipy)_2_(NO_3_)_2_]_2_ [67] (as determined by X-ray single-crystal diffraction) were isolated.

The IR spectrum of complex **7** is similar to related complex **4**; no significant changes could be found. However, the elemental analysis data indicated a 3:2 ratio of M:L instead of the 4:2 ratio found for complexes **3**–**6**.

**Figure 5:**
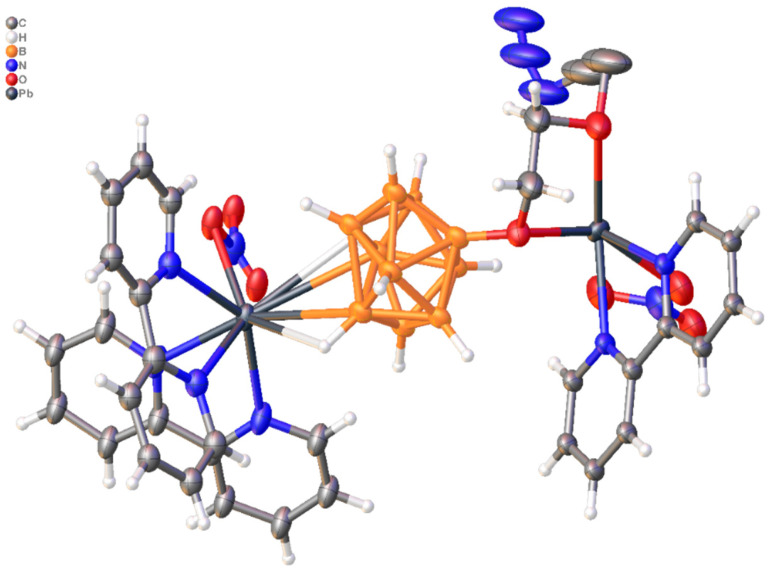
Structure of complex [(Pb(bipy)NO_3_)(Pb(bipy)_2_NO_3_)(B_10_H_9_O(CH_2_)_2_O(CH_2_)_2_N_3_)]·CH_3_CN·H_2_O (**7**·0.5CH_3_CN·0.125H_2_O). Thermal ellipsoids are shown with 30% probability level.

The crystallographically independent part of the monoclinic unit cell of complex **7** 0.5CH_3_CN·0.125H_2_O contains the binuclear lead(II) complex [(Pb(bipy)NO_3_)(Pb(bipy)_2_NO_3_)(B_10_H_9_O(CH_2_)_2_O(CH_2_)_2_N_3_)] (Figure 5). The terminal fragment (CH_2_)_2_N_3_ of the exo-polyhedral substituent is disordered into four positions with occupancies 0.3:0.3:0.2:0.2. The coordination environment of the Pb1 atom includes the B7B8 edge of the boron cage, two bipy molecules, and one nitrate anion. The Pb1–B bond lengths are 3.302(15) Å and 3.317(15) Å, whereas the Pb1–H bond lengths are 2.81(9) Å and 3.06(8) Å. The coordination environment of the Pb2 atom includes two oxygen atoms of the exo-polyhedral substituent (the length of the Pb2–O2 bond is significantly extended as compared to that of Pb2–O2; the Pb2–O1 bond length is 2.530(10) Å and the Pb2–O2 bond length is 2.882(13) Å).

**Figure 6:**
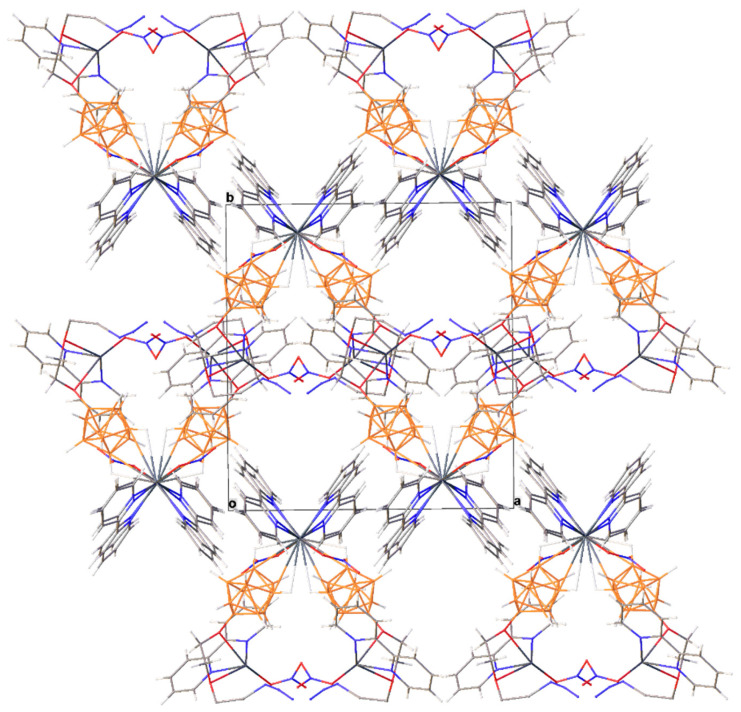
Packing diagram of complex **7**·0.5CH_3_CN·0.125H_2_O.

Complex **7**, similar to compound **4**, forms 1D polymer units due to π–π stacking interactions between bipy molecules of neighboring complexes. The units are interconnected through CH…HB contacts (Figure 6), forming channels parallel to axis c, which are filled with disordered solvent molecules.

In spite of the ability of lead(II) to form compounds with the N_3_ group coordinated by different methods [52], it seems that the softer boron cluster is preferable to form the coordination environment of the metal atom as compared to the azido group. We concluded that the N_3_ group is too hard to be coordinated by lead(II) in the presence of BH groups.

**Figure 7:**
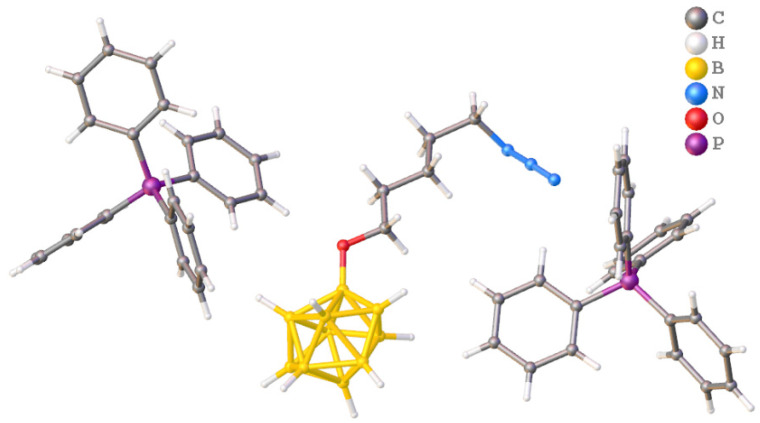
Structure of compound **2**.

Therefore, we tried to study cobalt(II) complexation because this metal is harder compared to lead(II). A number of cobalt(II) complexes with organoazides coordinated are known [57]; therefore, the formation of cobalt(II) complexes with boron clusters coordinated via the azide functional group could be expected. The experiments performed for compound **2** in the case of using Co(II) as a complexing agent revealed that the *closo*-decaborates with a pendant N_3_ functional group act as extremely weakly coordinating ligands in the absence of chelating ligands. In an attempt to obtain a Co(II) complex, when the *closo*-decaborate anion containing an azide pendent group was allowed to react with CoCl_2_ in the CH_3_CN/H_2_O system, the initial derivative of the *closo*-decaborate anion (compound **2**) was formed as a solid phase, the structure of which was determined by X-ray diffraction (Figure 7). According to the X-ray diffraction data, the structure of compound **2** consists of two Ph_4_P^+^ cations and the [2-B_10_H_9_O(CH_2_)_5_N_3_]^2−^ anion (Figure 7). The IR and ^11^B NMR data correlate with those reported [53].

**Scheme 4:**
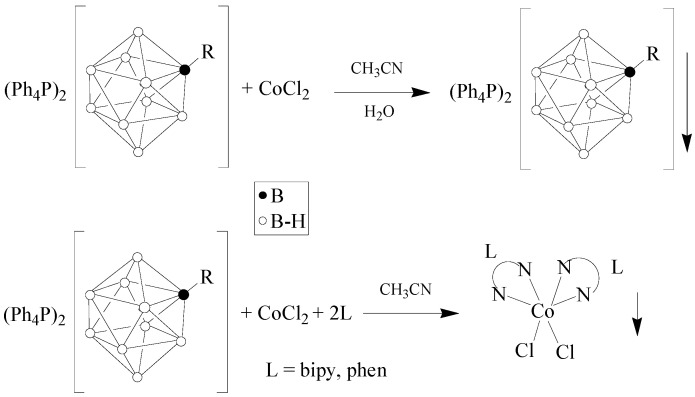
Cobalt(II) complexation reaction performed for compound **2**.

At the same time, when organic ligands L (bipy or phen) were used as additional ligands in the same reaction and the reaction was performed in CH_3_CN, heteroleptic cobalt(II) complexes [Co(bipy)_2_Cl_2_] or [Co(phen)_2_Cl_2_] were isolated, according to the single-crystal diffraction data [57,68]. In both cases, the starting compound (compound **2**) remained unreacted. It could be concluded that cobalt(II) seems to be too hard to form compounds with coordinated boron clusters and their derivatives. The reactions proceeded according to Scheme 4.

## 3. Experimental Section

### 3.1. Materials

Lead nitrate (99%), anhydrous cobalt chloride (98%), 2,2′-bipyridyl (bipy) (99%,), phenanthroline (phen) (99%), acetonitrile (for HPLC), tetrahydropyran (99%), 1,4-dioxane (99%), potassium hydroxide (95%), sodium azide (95%), and tetraphenylphosphonium chloride (99.9%) were purchased from Sigma-Aldrich (St. Louis, MO, USA) and used without additional purification. 

### 3.2. Syntheses

#### 3.2.1. Synthesis of Compounds **1** and **2**

Tetraphenylphosphonium 2-[2-(2-azidoetoxy)etoxy)]nonahydro-*closo*-decaborate (2-), (Ph_4_P)_2_[B_10_H_9_O(CH_2_)_2_O(CH_2_)_2_N_3_] (**1**) and tetraphenylphosphonium 2-[5-azidopentoxy]nonahydro-*closo*-decaborate (2-) (Ph_4_P)_2_[B_10_H_9_O(CH_2_)_5_N_3_] (**2**) were synthesized according to the previously reported method [53].

(Ph_4_P)_2_[B_10_H_9_O(CH_2_)_2_O(CH_2_)_2_N_3_] (**1**)

^11^B{H} NMR (CD_3_CN, ppm): −1.6 (s, 1B, B(2)), −3.1 (s, 1B, B(10)), −5.6 (s, 1B, B(1)), −23.7 (s, 4B, B(3,5,6,9)), −29.2 (s, 2B, B(7,8)), −34.1 (s, 1B, B(4)). ^1^H NMR (CD_3_CN, ppm): 7.95, 7.79, 7.71 (m, 40H, Ph_4_P^+^), 3.71 (t, 2H, OCH_2_CH_2_N_3_, *J* = 6.2 Hz), 3.25 (t, 2H, OCH_2_CH_2_OCH_2_, *J* = 5.3 Hz), 3.16 (t, 2H, OCH_2_CH_2_OCH_2_, *J* = 5.3 Hz), 3.08 (t, 2H, OCH_2_CH_2_N_3_, *J* = 6.2 Hz), 2.5…−0.5 (m, 9H, B-H). IR (selected bands, ν, cm^−1^): 2450 (ν(B–H)); 2108 (ν(N–N)).

(Ph_4_P)_2_[B_10_H_9_O(CH_2_)_5_N_3_] (**2**)

^11^B{H} NMR (CD_3_CN, ppm): −1.7 (s, 1B, B(2)), −2.7 (s, 1B, B(10)), −5.2 (s, 1B, B(1)), −23.8 (s, 4B, B(3,5,6,9)), −28.9 (s, 2B, B(7,8)), −33.6 (s, 1B, B(4)). ^1^H NMR (CD_3_CN, ppm): 7.96, 7.79, 7.70 (m, 40H, Ph_4_P^+^), 3.42 (t, 2H, OCH_2_CH_2_, *J* = 6.2 Hz), 3.05 (t, 2H, CH_2_CH_2_N_3_, *J* = 6.0 Hz), 1.86 (m, 2H, OCH_2_CH_2_CH_2_), 1.79 (m, 2H, CH_2_CH_2_N_3_), 1.32 (m, 2H, OCH_2_CH_2_CH_2_), 2.0…−0.5 (m, 9H, B-H). IR (selected bands, ν, cm^−1^): 2458 (ν(B–H)); 2112 (ν(N–N)).

#### 3.2.2. Synthesis of Compounds **3**–**7**

Pb(L)_2_[B_10_H_9_O(C_2_H_4_)O(C_2_H_4_)N_3_] (**3**:L = bipy, **4**:L = phen) and Pb_2_(L)_4_(NO_3_)_2_[B_10_H_9_O(CH_2_)_5_N_3_] (**5**:L = bipy, **6**:L = phen). 

Solid Pb(NO_3_)_2_ (0.2 mmol) was added to a solution of (Ph_4_P)_2_[An] (where [An]^2−^ = [B_10_H_9_O(CH_2_)_2_O(CH_2_)_2_N_3_]^2−^ (**1**) or [B_10_H_9_O(CH_2_)_5_N_3_]^2−^ (**2**)) (0.2 mmol) in acetonitrile (10 mL) and stirred for an hour. The unreacted Pb(NO_3_)_2_ was filtered off. A solution of ligand (phen, bipy) (0.4 mmol) in acetonitrile (10 mL) was added to the resulting yellow solution; the resulting mixture acquired a more saturated color. The formation of yellow crystals was observed after 20–25 min. Yield, 60–70%. Single crystals **4** and **6**·3CH_3_CN suitable for X-ray diffraction study were taken directly from the reaction solutions.

**(3)** Anal. calcd. for [Pb(bipy)_2_[B_10_H_9_O(CH_2_)_2_O(CH_2_)_2_N_3_], %: C, 37.6; H, 4.3; N, 12.8; B, 14.1; Pb, 27.0. Found for PbC_24_H_33_N_7_O_2_B_10_, %: C, 37.1; H, 4.2; N, 12.5; B, 14.2; Pb, 26.8. IR (ν, cm^−1^): ν(BH) 2464; ν(BH)_BHB_ 2359; ν(NN) 2093; ν(CH)_bipy_ 3068; ν(CN, CC)_bipy_ 1601, 1573, π(CH) 762. 

**(4)** Anal. calcd. for [Pb(phen)_2_[B_10_H_9_O(C_2_H_4_)O(C_2_H_4_)N_3_], %: C, 41.3; H, 4.1; N, 12.0; B, 13.3; Pb, 25.4. Found for PbC_28_H_33_N_7_O_2_B_10_, %: C, 41.7; H, 4.3; N, 12.1; B, 14.2; Pb, 25.3. IR (ν, cm^−1^): ν(BH) 2452; ν(BH)_BHB_ 2346; ν(N-N) 2095; ν(CH)_phen_ 3065; ν(CN, CC)_phen_ 1618, 1593, 1579; π(CH) 848, 720.

**(5)** Anal. calcd. for [Pb_2_(bipy)_4_(NO_3_)_2_[B_10_H_9_O(CH_2_)_5_N_3_], %: C, 38.4; H, 3.7; N, 12.9; B, 7.7; Pb, 29.5. Found for Pb_2_C_45_H_51_N_13_O_7_B_10_, %: C, 38.8; H, 3.4; N, 12.8; B, 7.8; Pb, 29.2. IR (ν, cm^−1^): ν(BH) 2452; ν(BH)_BHB_ 2346; ν(NN) 2095; ν(NO)_NO3_ 1500–1300; ν(CH)bipy 3069; 1605, 1572; π(CH) 760.

**(6)** Anal. calcd. for [Pb_2_(phen)_4_(NO_3_)_2_[B_10_H_9_O(CH_2_)_5_N_3_], %: C, 42.3; H, 3.4; N, 12.1; B, 7.2; Pb, 27.6. Found for Pb_2_C_53_H_51_N_13_O_7_B_10_, %: C, 42.3; H, 3.4; N, 12.1; B, 7.1; Pb, 27.7. IR (ν, cm^−1^): ν(BH) 2452; ν(BH)_BHB_ 2346; ν(NN) 2094; ν(NO)_NO3_ 1500–1300; ν(CN, CC)_phen_ 1620, 1595, 1578; π(CH) 851, 723.

[(Pb(bipy)NO_3_)(Pb(bipy)_2_NO_3_)(B_10_H_9_O(CH_2_)_2_O(CH_2_)_2_N_3_)] (**7**)

Pb(NO_3_)_2_ (0.2 mmol) was dissolved in water (5 mL) and the obtained solution was added to a solution of (Ph_4_P)_2_[An] (**2**) (0.2 mmol) in acetonitrile (10 mL). A solution of ligand (phen) (0.4 mmol) in acetonitrile (10 mL) was added to the resulting solution. The reaction mixtures of pale-yellow color were kept in air. The formation of yellow crystals was observed within 24 h. Yield, 57%. Single crystal **7**·CH_3_CN·H_2_O suitable for X-ray diffraction study was taken directly from the reaction solution.

**(7)** Anal. calcd. for [(Pb(bipy)NO_3_)(Pb(bipy)_2_NO_3_)(B_10_H_9_O(CH_2_)_2_O(CH_2_)_2_N_3_)], %: C, 34.9; H, 3.5; N, 6.0; B, 9.2; Pb, 35.4. Found for Pb_2_C_34_H_41_N_5_O_8_B_10_, %: C, 34.7; H, 3.4; N, 6.2; B, 9.0; Pb, 35.2. IR (ν, cm^−1^): ν(BH) 2465; ν(BH)_BHB_ 2353; ν(NN) 2094; ν(CH)_bipy_ 3067; ν(CN, CC)_bipy_ 1610, 1573, π(CH) 760.

#### 3.2.3. Cobalt(II) Complexation Using Compound **2**

CoCl_2_ (0.2 mmol) was dissolved in water (5 mL) and the obtained solution was added to a solution of (Ph_4_P)_2_[An] (**2**) (0.2 mmol) in acetonitrile (10 mL). The formation of gray crystal **2** was observed within 48 h. Yield, 78%. Single crystal **2** suitable for X-ray diffraction study was taken directly from the reaction solution. Data of physicochemical methods of analysis (NMR, IR) correlate with those indicated above for compound **2**.

CoCl_2_ (0.2 mmol) was dissolved in acetonitrile (5 mL) and the obtained blue solution was added to a solution of (Ph_4_P)_2_[An] (**2**) (0.2 mmol) in acetonitrile (10 mL). A solution of ligand (bipy or phen) (0.4 mmol) in acetonitrile (10 mL) was added to the resulting solution. The reaction mixtures were kept in air. Crystals [Co(bipy)_2_Cl_2_] and [Co(phen)_2_Cl_2_], respectively, were isolated from the reaction mixture after 48 h. According to the single-crystal diffraction data, the cell parameters of crystals correspond to known compounds [68,69]. Yield, 68% for [Co(bipy)_2_Cl_2_] and 72% for [Co(phen)_2_Cl_2_]. Compound **2** remained unreacted.

### 3.3. Methods

**Elemental analysis** of compounds **3**–**6** for carbon, hydrogen, and nitrogen was performed using a Carlo ErbaCHNS-3 FA 1108 automated elemental analyzer. Boron and metal content was determined on an iCAP 6300 Duo ICP emission spectrometer with inductively coupled plasma. Before the measurements, samples were dried in a vacuum to constant weight.

**IR spectra** of compounds were recorded on an Infralum FT-02 IR Fourier spectrometer (Lumex) in the range 4000–600 cm^−1^ at a resolution of 1 cm^−1^. Specimens were prepared as suspensions in Nujol mull (Aldrich), and NaCl glasses were used.

**^1^H and ^11^B NMR spectra** of CD_3_CN solutions of compounds **1** and **2** under study were recorded on a Bruker Avance II-300 spectrometer operating at a frequency of 300.3 and 96.32 MHz, respectively, using internal deuterium lock. Tetramethylsilane and boron trifluoride etherate were used as external references.

**Single-crystal X-ray diffraction** data for complexes **2**, **4**, **6**·3CH_3_CN, and **7**·0.5CH_3_CN·0.125H_2_O were collected on a three-circle Bruker D8 Venture diffractometer (*T* = 100 K, graphite monochromator, ω and φ scanning mode). The data were indexed and integrated using the SAINT program [70] and then scaled and corrected for absorption using the SADABS program [71]. For details, see Table S1 (Supplementary Materials).

The structure of **4** was determined by the intrinsic phasing method, and the structures of **2**, **6**, and **7** were solved by the charge flipping method implemented in Olex2 and refined by full-matrix least squares technique on *F*^2^ with anisotropic displacement parameters for non-hydrogen atoms. The hydrogen atoms were placed in calculated positions and refined within a riding model with fixed isotropic displacement parameters [*U*_iso_(H) = 1.5*U*_eq_(O), 1.5*U*_eq_(C) for the CH_3_ groups and 1.2*U*_eq_(C) for the other groups]. All calculations were carried out using the SHELXL program [72] and OLEX2 program package [73]. Errors in crystals **4** and **6** are probably due to the presence of a small twin component.

Crystallographic data for all investigated compounds have been deposited with the Cambridge Crystallographic Data Center, CCDC nos. 2307854–2307857. Copies of this information may be obtained free of charge from the Director, CCDC, 12 Union Road, Cambridge CB2 1EZ, UK (Fax: +44-1223-336033; e-mail: deposit@ccdc.cam.ac.uk or www.ccdc.cam.ac.uk; accessed on 12 December 2023).

## 4. Conclusions

Here, we studied lead(II) and cobalt(II) complexations of derivatives of the *closo*-decaborate anions [2-B_10_H_9_O(CH_2_)_2_O(CH_2_)_2_N_3_]^2−^ and [2-B_10_H_9_O(CH_2_)_5_N_3_]^2−^ containing pendant azido groups in the presence of 1,10-phenanthroline and 2,2′-bipyridyl. It was found that in acetonitrile, both derivatives form mononuclear lead(II) complexes [PbL_2_{An}] or binuclear lead(II) complexes [Pb_2_L_4_(NO_3_)_2_{An}] with the N_3_-substituted boron cluster coordinated via the BH groups or the BH group and the O atom of the substitute. In the CH_3_CN/water system, we succeeded in isolating complex [Pb_2_L_3_(NO_3_)_2_{An}] (An = [2-B_10_H_9_O(CH_2_)_5_N_3_]^2−^), where the N_3_-substituted boron cluster is coordinated by lead(II) via the BH groups and the O atom of the substitute. Complexes with a pendant N_3_ group involved in the metal coordination were not isolated. In the course of cobalt(II) complexation, starting compound (Ph_4_P)_2_[B_10_H_9_O(CH_2_)_5_N_3_] remained unreacted.

## Figures and Tables

**Table 1:** Selected bond lengths in the structure of **1**.

Bond	Length, Å	Bond	Length, Å
Pb–O1	2.443(7)	Pb2–O3	2.562(7)
Pb–N4	2.636(8)	Pb2–N11	2.616(8)
Pb–N5	2.571(9)	Pb2–N12	2.648(8)
Pb–N6	2.637(8)	Pb2–N13	2.662(8)
Pb–N7	2.572(8)	Pb2–N14	2.550(8)
Pb–B6	3.593(11)	Pb2–B16	3.524(13)
Pb–H6	3.7752(14)	Pb2–H16	3.6561(14)
Pb–B9	3.359(10)	Pb2–B19	3.358(12)
Pb–H9	3.2227(19)	Pb2–H19	3.2198(19)
Pb–B19	3.437(12)	Pb2–B9	3.567(11)
Pb–H19	2.9610(19)	Pb2–H9	3.044(2)
Pb–B20	3.463(13)	Pb2–B10	3.566(11)
Pb–H20	3.4576(14)	Pb2–H10	3.5091(14)

**Table 2:** Selected bond lengths in the structure of complex **6**·3CH_3_CN.

Bond	Length, Å	Bond	Length, Å
Pb1-O2	2.631(6)	Pb2-O5	2.683(6)
Pb1-O4	2.791(6)	Pb2-O6	2.695(6)
Pb1-N5	2.590(7)	Pb2-N10	2.608(7)
Pb1-N6	2.549(7)	Pb2-N11	2.624(7)
Pb1-N7	2.598(7)	Pb2-N12	2.520(7)
Pb1-N8	2.624(7)	Pb2-N13	2.610(7)
Pb1-B5	3.280(11)	Pb2-B3	3.338(11)
Pb1-H5	2.8317(11)	Pb2-H3	2.9056(8)
Pb1-B8	3.197(10)	Pb2-B6	3.192(10)
Pb1-H8	2.8195(10)	Pb2-H6	2.7792(10)
Pb1-B9	3.224(10)	Pb2-B7	3.268(10)
Pb1-H9	2.8843(12)	Pb2-H7	2.9327(12)

## Data Availability

Data are contained within the article and Supplementary Materials.

## Supplementary Materials

The following supporting information can be downloaded at: https://www.mdpi.com/article/10.3390/molecules28248073/s1, Table S1: Crystal data and structure refinement for compounds **2**–**7**, Crystallographic data files for compounds **2**–**7**.
