# Supplementary material for: Synthesis and Structures of Lead(II) Complexes with Substituted Derivatives of the Closo-Decaborate Anion with a Pendant N3 Group
Source: Molecules. 2023 Dec 13;28(24):8073. doi: 10.3390/molecules28248073 (PMC10746007; doi:10.3390/molecules28248073)

checkCIF/PLATON report

Structure factors have been supplied for datablock(s) mo_23kub134_0m_a

THIS REPORT IS FOR GUIDANCE ONLY. IF USED AS PART OF A REVIEW PROCEDURE FOR PUBLICATION, IT SHOULD NOT REPLACE THE EXPERTISE OF AN EXPERIENCED CRYSTALLOGRAPHIC REFEREE.

No syntax errors found. CIF dictionary Interpreting this report

Datablock: mo_23kub134_0m_a

Bond precision: C-C = 0.0059 A Wavelength=0.71073

Cell:	a=19.758(6)	b=13.276(2)	c=19.385(7)
	alpha=90	beta=96.157(14)	gamma=90
Temperature:	100 K		

	Calculated	Reported
Volume	5056(3)	5056(3)
Space group	P 21/c	P 1 21/c 1
Hall group	-P 2ybc	-P 2ybc
Moiety formula	2(C24 H20 P), C5 H19 B10 N3 O	2(C24 H20 P), C5 H19 B10 N3 O
Sum formula	C53 H59 B10 N3 O P2	C53 H59 B10 N3 O P2
Mr	924.07	924.07
Dx,g cm-3	1.214	1.214
Z	4	4
Mu (mm-1)	0.128	0.128
F000	1944.0	1944.0
F000'	1945.38	
h,k,lmax	26,17,25	26,17,25
Nref	12084	11803
Tmin,Tmax	0.982,0.987	0.589,0.746
Tmin'	0.975	

Correction method= # Reported T Limits: Tmin=0.589 Tmax=0.746
AbsCorr = MULTI-SCAN

Data completeness= 0.977 Theta(max)= 27.898

R(reflections)= 0.0789(6490) wR2(reflections)= 0.2253(11803)

S = 1.036 Npar= 622

The following ALERTS were generated. Each ALERT has the format
test-name_ALERT_alert-type_alert-level.
Click on the hyperlinks for more details of the test.

Alert level C

```
PLAT340_ALERT_3_C Low Bond Precision on  C-C Bonds ...............     0.0059 Ang.
PLAT767_ALERT_4_C INS Embedded LIST 6 Instruction Should be LIST 4     Please Check
PLAT905_ALERT_3_C Negative K value in the Analysis of Variance ...     -1.415 Report
PLAT911_ALERT_3_C Missing FCF Refl Between Thmin & STh/L=    0.600        151 Report
```

Alert level G

```
PLAT083_ALERT_2_G SHELXL Second Parameter in WGHT  Unusually Large       6.84 Why ?
PLAT883_ALERT_1_G No Info/Value for _atom_sites_solution_primary .     Please Do !
PLAT910_ALERT_3_G Missing # of FCF Reflection(s) Below Theta(Min).          1 Note
PLAT912_ALERT_4_G Missing # of FCF Reflections Above STh/L=  0.600        130 Note
PLAT941_ALERT_3_G Average HKL Measurement Multiplicity ...........        1.8 Low
PLAT953_ALERT_1_G Reported (CIF) and Actual (FCF) Hmax Differ by .          1 Units
PLAT978_ALERT_2_G Number C-C Bonds with Positive Residual Density.          3 Info
```

0 **ALERT level A** = Most likely a serious problem - resolve or explain
0 **ALERT level B** = A potentially serious problem, consider carefully
4 **ALERT level C** = Check. Ensure it is not caused by an omission or oversight
7 **ALERT level G** = General information/check it is not something unexpected

2 ALERT type 1 CIF construction/syntax error, inconsistent or missing data
2 ALERT type 2 Indicator that the structure model may be wrong or deficient
5 ALERT type 3 Indicator that the structure quality may be low
2 ALERT type 4 Improvement, methodology, query or suggestion
0 ALERT type 5 Informative message, check

It is advisable to attempt to resolve as many as possible of the alerts in all categories. Often the minor alerts point to easily fixed oversights, errors and omissions in your CIF or refinement strategy, so attention to these fine details can be worthwhile. In order to resolve some of the more serious problems it may be necessary to carry out additional measurements or structure refinements. However, the purpose of your study may justify the reported deviations and the more serious of these should normally be commented upon in the discussion or experimental section of a paper or in the "special_details" fields of the CIF. checkCIF was carefully designed to identify outliers and unusual parameters, but every test has its limitations and alerts that are not important in a particular case may appear. Conversely, the absence of alerts does not guarantee there are no aspects of the results needing attention. It is up to the individual to critically assess their own results and, if necessary, seek expert advice.

Publication of your CIF in IUCr journals

A basic structural check has been run on your CIF. These basic checks will be run on all CIFs submitted for publication in IUCr journals (*Acta Crystallographica*, *Journal of Applied Crystallography*, *Journal of Synchrotron Radiation*); however, if you intend to submit to *Acta Crystallographica Section C* or *E* or *IUCrData*, you should make sure that full publication checks are run on the final version of your CIF prior to submission.

Publication of your CIF in other journals

Please refer to the *Notes for Authors* of the relevant journal for any special instructions relating to CIF submission.

PLATON version of 06/07/2023; check.def file version of 30/06/2023

Datablock mo_23kub134_0m_a - ellipsoid plot

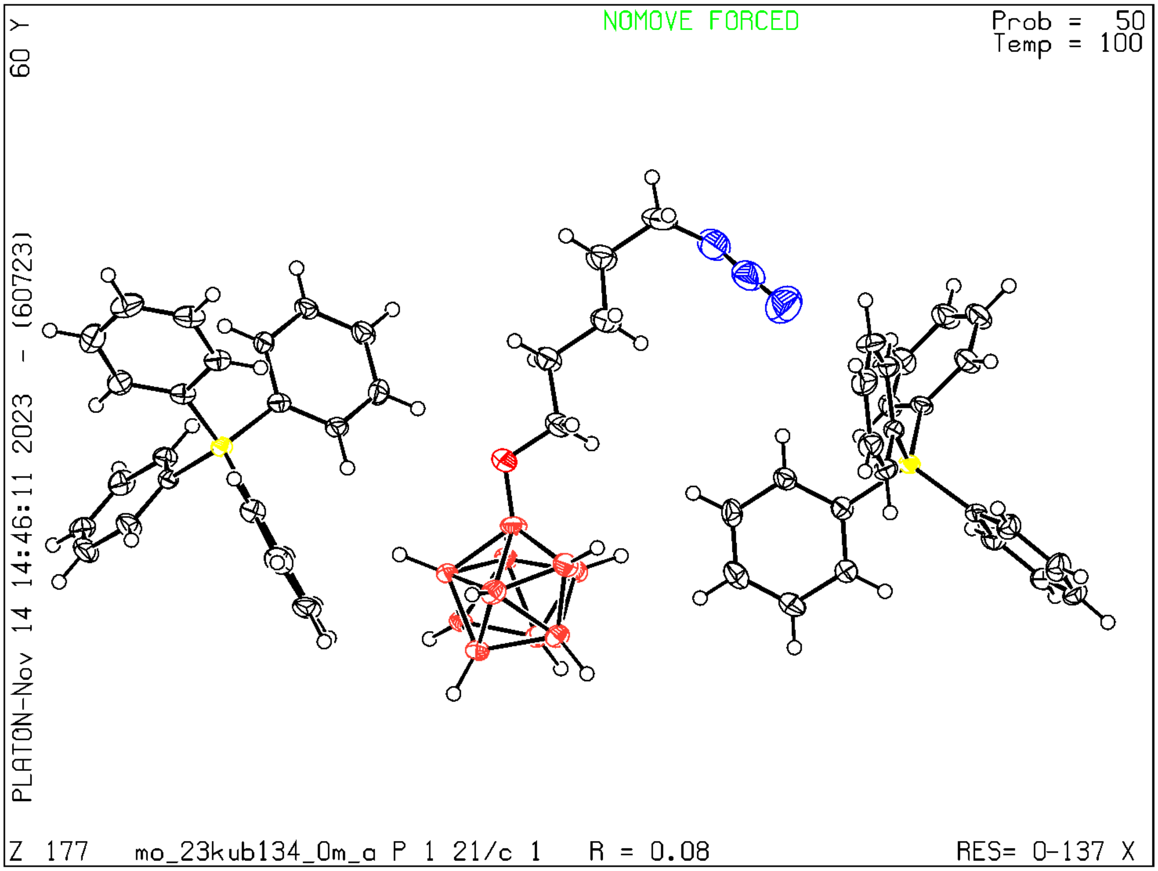

Supplement: Supplementary file 1 [file molecules-28-08073-s001.zip › 2_checkcif.pdf]
