# Supplementary material for: Synthesis and Structures of Lead(II) Complexes with Substituted Derivatives of the Closo-Decaborate Anion with a Pendant N3 Group
Source: Molecules. 2023 Dec 13;28(24):8073. doi: 10.3390/molecules28248073 (PMC10746007; doi:10.3390/molecules28248073)

checkCIF/PLATON report

Structure factors have been supplied for datablock(s) mo_23kub18_0m_a

THIS REPORT IS FOR GUIDANCE ONLY. IF USED AS PART OF A REVIEW PROCEDURE FOR PUBLICATION, IT SHOULD NOT REPLACE THE EXPERTISE OF AN EXPERIENCED CRYSTALLOGRAPHIC REFEREE.

No syntax errors found. CIF dictionary Interpreting this report

Datablock: mo_23kub18_0m_a

```
Bond precision:   C-C = 0.0163 A          Wavelength=0.71073

Cell:            a=11.273(5)       b=13.638(8)      c=22.354(14)
                 alpha=73.72(2)    beta=87.76(2)    gamma=88.16(2)
Temperature:     150 K
```

	Calculated	Reported
Volume	3296(3)	3296(3)
Space group	P -1	P -1
Hall group	-P 1	-P 1
Moiety formula	C28 H33 B10 N7 O2 Pb	C28 H33 B10 N7 O2 Pb
Sum formula	C28 H33 B10 N7 O2 Pb	C28 H33 B10 N7 O2 Pb
Mr	814.91	814.90
Dx,g cm-3	1.642	1.642
Z	4	4
Mu (mm-1)	5.159	5.160
F000	1592.0	1592.0
F000'	1579.11	
h,k,lmax	16,19,31	15,19,31
Nref	20163	18856
Tmin,Tmax	0.830,0.950	0.442,0.746
Tmin'	0.734	

```
Correction method= # Reported T Limits: Tmin=0.442 Tmax=0.746
AbsCorr = MULTI-SCAN

Data completeness= 0.935          Theta(max)= 30.530

R(reflections)= 0.0797( 11994)               wR2(reflections)= 0.2119( 18856)

S = 1.024                   Npar= 887
```

The following ALERTS were generated. Each ALERT has the format
test-name_ALERT_alert-type_alert-level.
Click on the hyperlinks for more details of the test.

Alert level B

```
PLAT097_ALERT_2_B Large Reported Max.  (Positive) Residual Density       8.67 eA-3
PLAT220_ALERT_2_B NonSolvent   Resd 1  N   Ueq(max)/Ueq(min) Range       10.0 Ratio
```

Alert level C

```
DIFMX02_ALERT_1_C  The maximum difference density is > 0.1*ZMAX*0.75
            The relevant atom site should be identified.
SHFSU01_ALERT_2_C  The absolute value of parameter shift to su ratio > 0.05
            Absolute value of the parameter shift to su ratio given   0.057
            Additional refinement cycles may be required.
PLAT029_ALERT_3_C _diffrn_measured_fraction_theta_full value Low .      0.978 Why?
PLAT080_ALERT_2_C Maximum Shift/Error ..........................         0.06 Why ?
PLAT094_ALERT_2_C Ratio of Maximum / Minimum Residual Density ....       2.30 Report
PLAT213_ALERT_2_C Atom N9A             has ADP max/min Ratio .....        3.3 oblate
PLAT213_ALERT_2_C Atom N9B             has ADP max/min Ratio .....        3.3 oblate
PLAT220_ALERT_2_C NonSolvent   Resd 1  C   Ueq(max)/Ueq(min) Range        5.4 Ratio
PLAT220_ALERT_2_C NonSolvent   Resd 2  C   Ueq(max)/Ueq(min) Range        3.4 Ratio
PLAT220_ALERT_2_C NonSolvent   Resd 2  N   Ueq(max)/Ueq(min) Range        3.6 Ratio
PLAT222_ALERT_3_C NonSolvent Resd 1  H   Uiso(max)/Uiso(min) Range        4.4 Ratio
PLAT234_ALERT_4_C Large Hirshfeld Difference C15      --C16      .       0.16 Ang.
PLAT342_ALERT_3_C Low Bond Precision on  C-C Bonds ..............     0.01634 Ang.
PLAT906_ALERT_3_C Large K Value in the Analysis of Variance ......      2.620 Check
PLAT911_ALERT_3_C Missing FCF Refl Between Thmin & STh/L=    0.600        263 Report
```

Alert level G

```
PLAT002_ALERT_2_G Number of Distance or Angle Restraints on AtSite         12 Note
PLAT072_ALERT_2_G SHELXL First  Parameter in WGHT  Unusually Large       0.10 Report
PLAT154_ALERT_1_G The s.u.'s on the Cell Angles are Equal ..(Note)       0.02 Degree
PLAT171_ALERT_4_G The CIF-Embedded .res File Contains EADP Records          7 Report
PLAT172_ALERT_4_G The CIF-Embedded .res File Contains DFIX Records         12 Report
PLAT300_ALERT_4_G Atom Site Occupancy of O4A       Constrained at       0.65 Check
PLAT300_ALERT_4_G Atom Site Occupancy of N8A       Constrained at       0.65 Check
PLAT300_ALERT_4_G Atom Site Occupancy of N9A       Constrained at       0.65 Check
PLAT300_ALERT_4_G Atom Site Occupancy of N10A      Constrained at       0.65 Check
PLAT300_ALERT_4_G Atom Site Occupancy of O4B       Constrained at       0.35 Check
PLAT300_ALERT_4_G Atom Site Occupancy of C28A      Constrained at       0.65 Check
PLAT300_ALERT_4_G Atom Site Occupancy of C29A      Constrained at       0.65 Check
PLAT300_ALERT_4_G Atom Site Occupancy of C30A      Constrained at       0.65 Check
PLAT300_ALERT_4_G Atom Site Occupancy of N8B       Constrained at       0.35 Check
PLAT300_ALERT_4_G Atom Site Occupancy of N9B       Constrained at       0.35 Check
PLAT300_ALERT_4_G Atom Site Occupancy of N10B      Constrained at       0.35 Check
PLAT300_ALERT_4_G Atom Site Occupancy of C28B      Constrained at       0.35 Check
PLAT300_ALERT_4_G Atom Site Occupancy of C29B      Constrained at       0.35 Check
PLAT300_ALERT_4_G Atom Site Occupancy of C30B      Constrained at       0.35 Check
PLAT300_ALERT_4_G Atom Site Occupancy of H27A      Constrained at       0.65 Check
PLAT300_ALERT_4_G Atom Site Occupancy of H27B      Constrained at       0.65 Check
PLAT300_ALERT_4_G Atom Site Occupancy of H28A      Constrained at       0.65 Check
PLAT300_ALERT_4_G Atom Site Occupancy of H28B      Constrained at       0.65 Check
```

```
PLAT300_ALERT_4_G Atom Site Occupancy of H29A       Constrained at       0.65 Check
PLAT300_ALERT_4_G Atom Site Occupancy of H29B       Constrained at       0.65 Check
PLAT300_ALERT_4_G Atom Site Occupancy of H30A       Constrained at       0.65 Check
PLAT300_ALERT_4_G Atom Site Occupancy of H30B       Constrained at       0.65 Check
PLAT300_ALERT_4_G Atom Site Occupancy of H27C       Constrained at       0.35 Check
PLAT300_ALERT_4_G Atom Site Occupancy of H27D       Constrained at       0.35 Check
PLAT300_ALERT_4_G Atom Site Occupancy of H28C       Constrained at       0.35 Check
PLAT300_ALERT_4_G Atom Site Occupancy of H28D       Constrained at       0.35 Check
PLAT300_ALERT_4_G Atom Site Occupancy of H29C       Constrained at       0.35 Check
PLAT300_ALERT_4_G Atom Site Occupancy of H29D       Constrained at       0.35 Check
PLAT300_ALERT_4_G Atom Site Occupancy of H30C       Constrained at       0.35 Check
PLAT300_ALERT_4_G Atom Site Occupancy of H30D       Constrained at       0.35 Check
PLAT301_ALERT_3_G Main Residue  Disorder ..............(Resd  1  )        15% Note
PLAT333_ALERT_2_G Large Aver C6-Ring C-C Dist C20      -C28      .       1.42 Ang.
PLAT722_ALERT_1_G Angle   Calc      109.00, Rep      110.10 Dev...       1.10 Degree
              O4A     -C28A    -H28B       1_555  1_555  1_555     #  310 Check
PLAT722_ALERT_1_G Angle   Calc      107.00, Rep      108.30 Dev...       1.30 Degree
              H28C    -C28B    -H28D       1_555  1_555  1_555     #  319 Check
PLAT794_ALERT_5_G Tentative Bond Valency for Pb1       (II)      .       1.76 Info
PLAT860_ALERT_3_G Number of Least-Squares Restraints .............         12 Note
PLAT870_ALERT_4_G ALERTS Related to Twinning Effects Suppressed ..          ! Info
PLAT883_ALERT_1_G No Info/Value for _atom_sites_solution_primary .     Please Do !
PLAT910_ALERT_3_G Missing # of FCF Reflection(s) Below Theta(Min).          2 Note
PLAT912_ALERT_4_G Missing # of FCF Reflections Above STh/L=  0.600       1016 Note
PLAT933_ALERT_2_G Number of HKL-OMIT Records in Embedded .res File          2 Note
PLAT941_ALERT_3_G Average HKL Measurement Multiplicity ...........        1.6 Low
```

---

   0 **ALERT level A** = Most likely a serious problem - resolve or explain
   2 **ALERT level B** = A potentially serious problem, consider carefully
  15 **ALERT level C** = Check. Ensure it is not caused by an omission or oversight
  47 **ALERT level G** = General information/check it is not something unexpected

   5 ALERT type 1 CIF construction/syntax error, inconsistent or missing data
  14 ALERT type 2 Indicator that the structure model may be wrong or deficient
   9 ALERT type 3 Indicator that the structure quality may be low
  35 ALERT type 4 Improvement, methodology, query or suggestion
   1 ALERT type 5 Informative message, check

It is advisable to attempt to resolve as many as possible of the alerts in all categories. Often the minor alerts point to easily fixed oversights, errors and omissions in your CIF or refinement strategy, so attention to these fine details can be worthwhile. In order to resolve some of the more serious problems it may be necessary to carry out additional measurements or structure refinements. However, the purpose of your study may justify the reported deviations and the more serious of these should normally be commented upon in the discussion or experimental section of a paper or in the "special_details" fields of the CIF. checkCIF was carefully designed to identify outliers and unusual parameters, but every test has its limitations and alerts that are not important in a particular case may appear. Conversely, the absence of alerts does not guarantee there are no aspects of the results needing attention. It is up to the individual to critically assess their own results and, if necessary, seek expert advice.

Publication of your CIF in IUCr journals

A basic structural check has been run on your CIF. These basic checks will be run on all CIFs submitted for publication in IUCr journals (*Acta Crystallographica*, *Journal of Applied Crystallography*, *Journal of Synchrotron Radiation*); however, if you intend to submit to *Acta Crystallographica Section C* or *E* or *IUCrData*, you should make sure that full publication checks are run on the final version of your CIF prior to submission.

Publication of your CIF in other journals

Please refer to the *Notes for Authors* of the relevant journal for any special instructions relating to CIF submission.

PLATON version of 06/07/2023; check.def file version of 30/06/2023

Datablock mo_23kub18_0m_a - ellipsoid plot

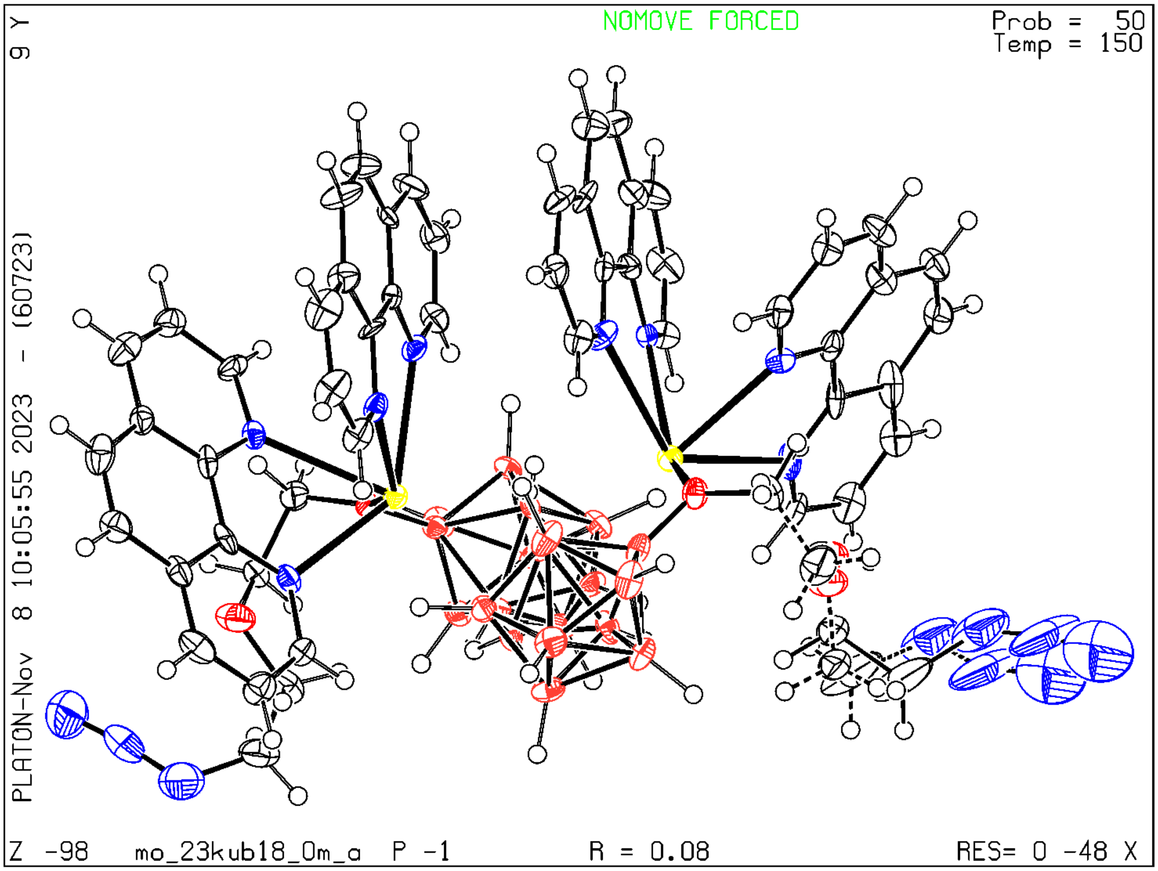

Supplement: Supplementary file 1 [file molecules-28-08073-s001.zip › 4_cifreport.pdf]
