# Supplementary material for: Synthesis and Structures of Lead(II) Complexes with Substituted Derivatives of the Closo-Decaborate Anion with a Pendant N3 Group
Source: Molecules. 2023 Dec 13;28(24):8073. doi: 10.3390/molecules28248073 (PMC10746007; doi:10.3390/molecules28248073)

checkCIF/PLATON report

Structure factors have been supplied for datablock(s) mo_22kub294_0m_a

THIS REPORT IS FOR GUIDANCE ONLY. IF USED AS PART OF A REVIEW PROCEDURE FOR PUBLICATION, IT SHOULD NOT REPLACE THE EXPERTISE OF AN EXPERIENCED CRYSTALLOGRAPHIC REFEREE.

No syntax errors found. CIF dictionary Interpreting this report

Datablock: mo_22kub294_0m_a

Bond precision: C-C = 0.0135 A Wavelength=0.71073

Cell:	a=9.377(3)	b=17.981(6)	c=19.298(10)
	alpha=99.786(19)	beta=95.463(18)	gamma=97.987(9)
Temperature:	100 K		

	Calculated	Reported
Volume	3152(2)	3152(2)
Space group	P -1	P -1
Hall group	-P 1	-P 1
Moiety formula	C53 H51 B10 N13 O7 Pb2, 3(C2 H3 N)	C53 H51 B10 N13 O7 Pb2, 3(C2 H3 N)
Sum formula	C59 H60 B10 N16 O7 Pb2	C59 H60 B10 N16 O7 Pb2
Mr	1627.73	1627.71
Dx,g cm-3	1.715	1.715
Z	2	2
Mu (mm-1)	5.401	5.402
F000	1592.0	1592.0
F000'	1579.20	
h,k,lmax	11,21,23	11,21,23
Nref	11231	10974
Tmin,Tmax	0.806,0.947	0.603,0.745
Tmin'	0.806	

Correction method= # Reported T Limits: Tmin=0.603 Tmax=0.745
AbsCorr = MULTI-SCAN

Data completeness= 0.977 Theta(max)= 25.103

R(reflections)= 0.0517(8876) wR2(reflections)= 0.1127(10974)

S = 1.075 Npar= 838

The following ALERTS were generated. Each ALERT has the format
test-name_ALERT_alert-type_alert-level.
Click on the hyperlinks for more details of the test.

Alert level B

```
PLAT971_ALERT_2_B Check Calcd Resid. Dens.  1.67Ang From C51           3.14 eA-3
```

Alert level C

```
PLAT029_ALERT_3_C _diffrn_measured_fraction_theta_full value Low .      0.977 Why?
PLAT094_ALERT_2_C Ratio of Maximum / Minimum Residual Density ....       2.33 Report
PLAT213_ALERT_2_C Atom O2              has ADP max/min Ratio .....        3.2 prolat
PLAT213_ALERT_2_C Atom C7              has ADP max/min Ratio .....        3.5 prolat
PLAT220_ALERT_2_C NonSolvent   Resd 1  N   Ueq(max)/Ueq(min) Range        3.7 Ratio
PLAT241_ALERT_2_C High   'MainMol' Ueq as Compared to Neighbors of         N1 Check
PLAT242_ALERT_2_C Low    'MainMol' Ueq as Compared to Neighbors of         N2 Check
PLAT342_ALERT_3_C Low Bond Precision on  C-C Bonds ...............    0.01355 Ang.
PLAT751_ALERT_4_C Bond    Calc     2.83000, Rep  2.8317(11) ......  Senseless s.u.
              PB2      -H5              1_555   1_555 ........     #    8 Check
PLAT751_ALERT_4_C Bond    Calc     2.82000, Rep  2.8195(10) ......  Senseless s.u.
              PB2      -H8A             1_555   1_555 ........     #   10 Check
PLAT751_ALERT_4_C Bond    Calc     2.88000, Rep  2.8843(12) ......  Senseless s.u.
              PB2      -H9              1_555   1_555 ........     #   12 Check
PLAT751_ALERT_4_C Bond    Calc     2.91000, Rep   2.9056(8) ......  Senseless s.u.
              PB1      -H3              1_555   1_555 ........     #   71 Check
PLAT751_ALERT_4_C Bond    Calc     2.78000, Rep  2.7792(10) ......  Senseless s.u.
              PB1      -H6A             1_555   1_555 ........     #   73 Check
PLAT751_ALERT_4_C Bond    Calc     2.93000, Rep  2.9327(12) ......  Senseless s.u.
              PB1      -H7A             1_555   1_555 ........     #   75 Check
PLAT751_ALERT_4_C Bond    Calc     1.12000, Rep   1.120(10) ......  Senseless s.u.
              B3       -H3              1_555   1_555 ........     #  155 Check
PLAT751_ALERT_4_C Bond    Calc     1.12000, Rep   1.119(10) ......  Senseless s.u.
              B5       -H5              1_555   1_555 ........     #  163 Check
PLAT751_ALERT_4_C Bond    Calc     1.12000, Rep   1.119(10) ......  Senseless s.u.
              B6       -H6A             1_555   1_555 ........     #  166 Check
PLAT751_ALERT_4_C Bond    Calc     1.12000, Rep   1.119(10) ......  Senseless s.u.
              B7       -H7A             1_555   1_555 ........     #  170 Check
PLAT751_ALERT_4_C Bond    Calc     1.12000, Rep   1.118(10) ......  Senseless s.u.
              B8       -H8A             1_555   1_555 ........     #  173 Check
PLAT751_ALERT_4_C Bond    Calc     1.12000, Rep   1.120(10) ......  Senseless s.u.
              B9       -H9              1_555   1_555 ........     #  176 Check
PLAT752_ALERT_4_C Angle   Calc       75.00, Rep   74.82(14) ......  Senseless s.u.
              O2      -PB2     -H5         1_555  1_555  1_555     #    3 Check
PLAT752_ALERT_4_C Angle   Calc      138.00, Rep  137.53(14) ......  Senseless s.u.
              O2      -PB2     -H8A        1_555  1_555  1_555     #    5 Check
PLAT752_ALERT_4_C Angle   Calc       86.00, Rep   86.29(13) ......  Senseless s.u.
              O2      -PB2     -H9         1_555  1_555  1_555     #    7 Check
PLAT752_ALERT_4_C Angle   Calc       98.00, Rep   97.94(14) ......  Senseless s.u.
              O4      -PB2     -H5         1_555  1_555  1_555     #    9 Check
PLAT752_ALERT_4_C Angle   Calc      133.00, Rep  133.34(12) ......  Senseless s.u.
              O4      -PB2     -H8A        1_555  1_555  1_555     #   11 Check
PLAT752_ALERT_4_C Angle   Calc       62.00, Rep   62.11(12) ......  Senseless s.u.
              O4      -PB2     -H9         1_555  1_555  1_555     #   13 Check
PLAT752_ALERT_4_C Angle   Calc      143.00, Rep  143.11(15) ......  Senseless s.u.
              N5      -PB2     -H5         1_555  1_555  1_555     #   19 Check
```

```
PLAT752_ALERT_4_C Angle   Calc       92.00, Rep   91.67(15) ......  Senseless s.u.
              N5      -PB2     -H8A        1_555  1_555  1_555     #   21 Check
PLAT752_ALERT_4_C Angle   Calc       86.00, Rep   85.95(15) ......  Senseless s.u.
              N5      -PB2     -H9         1_555  1_555  1_555     #   23 Check
PLAT752_ALERT_4_C Angle   Calc      150.00, Rep  149.74(16) ......  Senseless s.u.
              N6      -PB2     -H5         1_555  1_555  1_555     #   30 Check
PLAT752_ALERT_4_C Angle   Calc      144.00, Rep  144.06(16) ......  Senseless s.u.
              N6      -PB2     -H8A        1_555  1_555  1_555     #   32 Check
PLAT752_ALERT_4_C Angle   Calc      129.00, Rep  128.88(14) ......  Senseless s.u.
              N6      -PB2     -H9         1_555  1_555  1_555     #   34 Check
PLAT752_ALERT_4_C Angle   Calc      113.00, Rep  112.85(15) ......  Senseless s.u.
              N7      -PB2     -H5         1_555  1_555  1_555     #   39 Check
PLAT752_ALERT_4_C Angle   Calc       71.00, Rep   71.50(15) ......  Senseless s.u.
              N7      -PB2     -H8A        1_555  1_555  1_555     #   41 Check
PLAT752_ALERT_4_C Angle   Calc      140.00, Rep  139.80(15) ......  Senseless s.u.
              N7      -PB2     -H9         1_555  1_555  1_555     #   43 Check
PLAT752_ALERT_4_C Angle   Calc       79.00, Rep   79.37(16) ......  Senseless s.u.
              N8      -PB2     -H5         1_555  1_555  1_555     #   47 Check
PLAT752_ALERT_4_C Angle   Calc      100.00, Rep  100.43(15) ......  Senseless s.u.
              N8      -PB2     -H8A        1_555  1_555  1_555     #   49 Check
PLAT752_ALERT_4_C Angle   Calc      140.00, Rep  139.81(16) ......  Senseless s.u.
              N8      -PB2     -H9         1_555  1_555  1_555     #   51 Check
PLAT752_ALERT_4_C Angle   Calc       19.00, Rep   19.36(18) ......  Senseless s.u.
              H5      -PB2     -B5         1_555  1_555  1_555     #   52 Check
PLAT752_ALERT_4_C Angle   Calc       50.00, Rep   49.86(18) ......  Senseless s.u.
              H5      -PB2     -B8         1_555  1_555  1_555     #   53 Check
PLAT752_ALERT_4_C Angle   Calc       49.00, Rep   49.43(18) ......  Senseless s.u.
              H5      -PB2     -B9         1_555  1_555  1_555     #   54 Check
PLAT752_ALERT_4_C Angle   Calc       62.00, Rep    61.71(3) ......  Senseless s.u.
              H5      -PB2     -H9         1_555  1_555  1_555     #   55 Check
PLAT752_ALERT_4_C Angle   Calc       49.00, Rep   49.03(18) ......  Senseless s.u.
              H8A     -PB2     -B5         1_555  1_555  1_555     #   58 Check
PLAT752_ALERT_4_C Angle   Calc       63.00, Rep    62.83(3) ......  Senseless s.u.
              H8A     -PB2     -H5         1_555  1_555  1_555     #   59 Check
PLAT752_ALERT_4_C Angle   Calc       20.00, Rep   20.19(17) ......  Senseless s.u.
              H8A     -PB2     -B8         1_555  1_555  1_555     #   60 Check
PLAT752_ALERT_4_C Angle   Calc       53.00, Rep   53.02(19) ......  Senseless s.u.
              H8A     -PB2     -B9         1_555  1_555  1_555     #   61 Check
PLAT752_ALERT_4_C Angle   Calc       72.00, Rep    71.59(3) ......  Senseless s.u.
              H8A     -PB2     -H9         1_555  1_555  1_555     #   62 Check
PLAT752_ALERT_4_C Angle   Calc       48.00, Rep   48.49(17) ......  Senseless s.u.
              H9      -PB2     -B5         1_555  1_555  1_555     #   64 Check
PLAT752_ALERT_4_C Angle   Calc       53.00, Rep   53.09(17) ......  Senseless s.u.
              H9      -PB2     -B8         1_555  1_555  1_555     #   65 Check
PLAT752_ALERT_4_C Angle   Calc       20.00, Rep   20.16(18) ......  Senseless s.u.
              H9      -PB2     -B9         1_555  1_555  1_555     #   66 Check
PLAT752_ALERT_4_C Angle   Calc      132.00, Rep  132.27(13) ......  Senseless s.u.
              O5      -PB1     -H3         1_555  1_555  1_555     #  158 Check
PLAT752_ALERT_4_C Angle   Calc       70.00, Rep   69.77(13) ......  Senseless s.u.
              O5      -PB1     -H6A        1_555  1_555  1_555     #  160 Check
PLAT752_ALERT_4_C Angle   Calc      111.00, Rep  110.84(14) ......  Senseless s.u.
              O5      -PB1     -H7A        1_555  1_555  1_555     #  162 Check
PLAT752_ALERT_4_C Angle   Calc      153.00, Rep  153.30(13) ......  Senseless s.u.
              O6      -PB1     -H3         1_555  1_555  1_555     #  164 Check
PLAT752_ALERT_4_C Angle   Calc      104.00, Rep  104.14(12) ......  Senseless s.u.
              O6      -PB1     -H6A        1_555  1_555  1_555     #  166 Check
PLAT752_ALERT_4_C Angle   Calc       94.00, Rep   93.49(13) ......  Senseless s.u.
```

```
              O6      -PB1     -H7A        1_555  1_555  1_555     #  168 Check
PLAT752_ALERT_4_C Angle   Calc      103.00, Rep  102.54(16) ......  Senseless s.u.
              N10     -PB1     -H3         1_555  1_555  1_555     #  174 Check
PLAT752_ALERT_4_C Angle   Calc      147.00, Rep  146.50(15) ......  Senseless s.u.
              N10     -PB1     -H6A        1_555  1_555  1_555     #  176 Check
PLAT752_ALERT_4_C Angle   Calc       76.00, Rep   75.99(16) ......  Senseless s.u.
              N10     -PB1     -H7A        1_555  1_555  1_555     #  178 Check
PLAT752_ALERT_4_C Angle   Calc       69.00, Rep   69.39(16) ......  Senseless s.u.
              N11     -PB1     -H3         1_555  1_555  1_555     #  182 Check
PLAT752_ALERT_4_C Angle   Calc      127.00, Rep  127.43(15) ......  Senseless s.u.
              N11     -PB1     -H6A        1_555  1_555  1_555     #  184 Check
PLAT752_ALERT_4_C Angle   Calc      105.00, Rep  104.66(16) ......  Senseless s.u.
              N11     -PB1     -H7A        1_555  1_555  1_555     #  186 Check
PLAT752_ALERT_4_C Angle   Calc      136.00, Rep  135.86(17) ......  Senseless s.u.
              N12     -PB1     -H3         1_555  1_555  1_555     #  193 Check
PLAT752_ALERT_4_C Angle   Calc      128.00, Rep  128.22(15) ......  Senseless s.u.
              N12     -PB1     -H6A        1_555  1_555  1_555     #  195 Check
PLAT752_ALERT_4_C Angle   Calc      157.00, Rep  157.19(16) ......  Senseless s.u.
              N12     -PB1     -H7A        1_555  1_555  1_555     #  197 Check
PLAT752_ALERT_4_C Angle   Calc       85.00, Rep   85.11(16) ......  Senseless s.u.
              N13     -PB1     -H3         1_555  1_555  1_555     #  202 Check
PLAT752_ALERT_4_C Angle   Calc       73.00, Rep   72.68(15) ......  Senseless s.u.
              N13     -PB1     -H6A        1_555  1_555  1_555     #  204 Check
PLAT752_ALERT_4_C Angle   Calc      138.00, Rep  138.10(16) ......  Senseless s.u.
              N13     -PB1     -H7A        1_555  1_555  1_555     #  206 Check
PLAT752_ALERT_4_C Angle   Calc       19.00, Rep   19.10(18) ......  Senseless s.u.
              H3      -PB1     -B3         1_555  1_555  1_555     #  207 Check
PLAT752_ALERT_4_C Angle   Calc       50.00, Rep   49.88(18) ......  Senseless s.u.
              H3      -PB1     -B6         1_555  1_555  1_555     #  208 Check
PLAT752_ALERT_4_C Angle   Calc       49.00, Rep     48.7(2) ......  Senseless s.u.
              H3      -PB1     -B7         1_555  1_555  1_555     #  209 Check
PLAT752_ALERT_4_C Angle   Calc       61.00, Rep    60.59(2) ......  Senseless s.u.
              H3      -PB1     -H7A        1_555  1_555  1_555     #  210 Check
PLAT752_ALERT_4_C Angle   Calc       49.00, Rep   49.19(17) ......  Senseless s.u.
              H6A     -PB1     -B3         1_555  1_555  1_555     #  213 Check
PLAT752_ALERT_4_C Angle   Calc       63.00, Rep    63.16(2) ......  Senseless s.u.
              H6A     -PB1     -H3         1_555  1_555  1_555     #  214 Check
PLAT752_ALERT_4_C Angle   Calc       20.00, Rep   20.11(17) ......  Senseless s.u.
              H6A     -PB1     -B6         1_555  1_555  1_555     #  215 Check
PLAT752_ALERT_4_C Angle   Calc       52.00, Rep   52.37(16) ......  Senseless s.u.
              H6A     -PB1     -B7         1_555  1_555  1_555     #  216 Check
PLAT752_ALERT_4_C Angle   Calc       71.00, Rep    70.63(3) ......  Senseless s.u.
              H6A     -PB1     -H7A        1_555  1_555  1_555     #  217 Check
PLAT752_ALERT_4_C Angle   Calc       48.00, Rep   47.71(19) ......  Senseless s.u.
              H7A     -PB1     -B3         1_555  1_555  1_555     #  219 Check
PLAT752_ALERT_4_C Angle   Calc       52.00, Rep   52.26(18) ......  Senseless s.u.
              H7A     -PB1     -B6         1_555  1_555  1_555     #  220 Check
PLAT752_ALERT_4_C Angle   Calc       20.00, Rep   19.87(17) ......  Senseless s.u.
              H7A     -PB1     -B7         1_555  1_555  1_555     #  221 Check
PLAT752_ALERT_4_C Angle   Calc       58.00, Rep     58.1(5) ......  Senseless s.u.
              PB1     -B3      -H3         1_555  1_555  1_555     #  369 Check
PLAT752_ALERT_4_C Angle   Calc      118.00, Rep    118.4(9) ......  Senseless s.u.
              B1      -B3      -H3         1_555  1_555  1_555     #  372 Check
PLAT752_ALERT_4_C Angle   Calc      130.00, Rep    130.1(9) ......  Senseless s.u.
              B2      -B3      -H3         1_555  1_555  1_555     #  377 Check
PLAT752_ALERT_4_C Angle   Calc      131.00, Rep    131.2(8) ......  Senseless s.u.
              B4      -B3      -H3         1_555  1_555  1_555     #  381 Check
```

```
PLAT752_ALERT_4_C Angle   Calc      120.00, Rep    120.0(8) ......  Senseless s.u.
              B6      -B3      -H3         1_555  1_555  1_555     #  383 Check
PLAT752_ALERT_4_C Angle   Calc      120.00, Rep    120.3(8) ......  Senseless s.u.
              B7      -B3      -H3         1_555  1_555  1_555     #  387 Check
PLAT752_ALERT_4_C Angle   Calc       57.00, Rep     57.1(5) ......  Senseless s.u.
              PB2     -B5      -H5         1_555  1_555  1_555     #  405 Check
PLAT752_ALERT_4_C Angle   Calc      119.00, Rep    119.1(9) ......  Senseless s.u.
              B1      -B5      -H5         1_555  1_555  1_555     #  409 Check
PLAT752_ALERT_4_C Angle   Calc      131.00, Rep    131.3(8) ......  Senseless s.u.
              B2      -B5      -H5         1_555  1_555  1_555     #  413 Check
PLAT752_ALERT_4_C Angle   Calc      131.00, Rep    130.6(8) ......  Senseless s.u.
              B4      -B5      -H5         1_555  1_555  1_555     #  416 Check
PLAT752_ALERT_4_C Angle   Calc      120.00, Rep    120.1(9) ......  Senseless s.u.
              B8      -B5      -H5         1_555  1_555  1_555     #  420 Check
PLAT752_ALERT_4_C Angle   Calc      120.00, Rep    119.9(8) ......  Senseless s.u.
              B9      -B5      -H5         1_555  1_555  1_555     #  424 Check
PLAT752_ALERT_4_C Angle   Calc       59.00, Rep     58.6(4) ......  Senseless s.u.
              PB1     -B6      -H6A        1_555  1_555  1_555     #  426 Check
PLAT752_ALERT_4_C Angle   Calc      121.00, Rep    120.7(8) ......  Senseless s.u.
              B2      -B6      -H6A        1_555  1_555  1_555     #  429 Check
PLAT752_ALERT_4_C Angle   Calc      121.00, Rep    121.1(8) ......  Senseless s.u.
              B3      -B6      -H6A        1_555  1_555  1_555     #  432 Check
PLAT752_ALERT_4_C Angle   Calc      131.00, Rep    130.8(8) ......  Senseless s.u.
              B7      -B6      -H6A        1_555  1_555  1_555     #  435 Check
PLAT752_ALERT_4_C Angle   Calc      131.00, Rep    131.0(8) ......  Senseless s.u.
              B9      -B6      -H6A        1_555  1_555  1_555     #  439 Check
PLAT752_ALERT_4_C Angle   Calc      117.00, Rep    117.1(8) ......  Senseless s.u.
              B10     -B6      -H6A        1_555  1_555  1_555     #  444 Check
PLAT752_ALERT_4_C Angle   Calc       63.00, Rep     62.9(5) ......  Senseless s.u.
              PB1     -B7      -H7A        1_555  1_555  1_555     #  447 Check
PLAT752_ALERT_4_C Angle   Calc      120.00, Rep    119.8(8) ......  Senseless s.u.
              B3      -B7      -H7A        1_555  1_555  1_555     #  451 Check
PLAT752_ALERT_4_C Angle   Calc      120.00, Rep    119.5(8) ......  Senseless s.u.
              B4      -B7      -H7A        1_555  1_555  1_555     #  455 Check
PLAT752_ALERT_4_C Angle   Calc      131.00, Rep    131.1(9) ......  Senseless s.u.
              B6      -B7      -H7A        1_555  1_555  1_555     #  458 Check
PLAT752_ALERT_4_C Angle   Calc      131.00, Rep    131.3(9) ......  Senseless s.u.
              B8      -B7      -H7A        1_555  1_555  1_555     #  461 Check
PLAT752_ALERT_4_C Angle   Calc      118.00, Rep    118.2(9) ......  Senseless s.u.
              B10     -B7      -H7A        1_555  1_555  1_555     #  466 Check
PLAT752_ALERT_4_C Angle   Calc       61.00, Rep     60.5(4) ......  Senseless s.u.
              PB2     -B8      -H8A        1_555  1_555  1_555     #  468 Check
PLAT752_ALERT_4_C Angle   Calc      120.00, Rep    120.2(8) ......  Senseless s.u.
              B4      -B8      -H8A        1_555  1_555  1_555     #  471 Check
PLAT752_ALERT_4_C Angle   Calc      120.00, Rep    120.4(8) ......  Senseless s.u.
              B5      -B8      -H8A        1_555  1_555  1_555     #  476 Check
PLAT752_ALERT_4_C Angle   Calc      131.00, Rep    130.8(8) ......  Senseless s.u.
              B7      -B8      -H8A        1_555  1_555  1_555     #  479 Check
PLAT752_ALERT_4_C Angle   Calc      131.00, Rep    131.1(8) ......  Senseless s.u.
              B9      -B8      -H8A        1_555  1_555  1_555     #  482 Check
PLAT752_ALERT_4_C Angle   Calc      117.00, Rep    116.8(8) ......  Senseless s.u.
              B10     -B8      -H8A        1_555  1_555  1_555     #  487 Check
PLAT752_ALERT_4_C Angle   Calc       63.00, Rep     62.5(5) ......  Senseless s.u.
              PB2     -B9      -H9         1_555  1_555  1_555     #  489 Check
PLAT752_ALERT_4_C Angle   Calc      119.00, Rep    119.3(8) ......  Senseless s.u.
              B2      -B9      -H9         1_555  1_555  1_555     #  494 Check
PLAT752_ALERT_4_C Angle   Calc      120.00, Rep    119.8(8) ......  Senseless s.u.
```

```
              B5      -B9      -H9         1_555  1_555  1_555     #  498 Check
PLAT752_ALERT_4_C Angle   Calc      131.00, Rep    131.1(8) ......  Senseless s.u.
              B6      -B9      -H9         1_555  1_555  1_555     #  501 Check
PLAT752_ALERT_4_C Angle   Calc      132.00, Rep    131.9(8) ......  Senseless s.u.
              B8      -B9      -H9         1_555  1_555  1_555     #  503 Check
PLAT752_ALERT_4_C Angle   Calc      119.00, Rep    119.0(8) ......  Senseless s.u.
              B10     -B9      -H9         1_555  1_555  1_555     #  509 Check
PLAT767_ALERT_4_C INS Embedded LIST 6 Instruction Should be LIST 4     Please Check
PLAT906_ALERT_3_C Large K Value in the Analysis of Variance ......      4.866 Check
PLAT911_ALERT_3_C Missing FCF Refl Between Thmin & STh/L=    0.597        254 Report
PLAT971_ALERT_2_C Check Calcd Resid. Dens.  0.98Ang From Pb2           1.66 eA-3
PLAT971_ALERT_2_C Check Calcd Resid. Dens.  0.95Ang From Pb2           1.54 eA-3
PLAT971_ALERT_2_C Check Calcd Resid. Dens.  0.92Ang From Pb1           1.53 eA-3
PLAT972_ALERT_2_C Check Calcd Resid. Dens.  1.77Ang From C14          -1.59 eA-3
```

Alert level G

```
PLAT083_ALERT_2_G SHELXL Second Parameter in WGHT  Unusually Large      23.74 Why ?
PLAT171_ALERT_4_G The CIF-Embedded .res File Contains EADP Records          1 Report
PLAT432_ALERT_2_G Short Inter X...Y Contact  O7       ..C44      .       2.99 Ang.
                                                  -x,1-y,-z  =      2_565 Check
PLAT790_ALERT_4_G Centre of Gravity not Within Unit Cell: Resd.  #          2 Note
              C2 H3 N
PLAT790_ALERT_4_G Centre of Gravity not Within Unit Cell: Resd.  #          4 Note
              C2 H3 N
PLAT794_ALERT_5_G Tentative Bond Valency for Pb1       (II)      .       1.99 Info
PLAT794_ALERT_5_G Tentative Bond Valency for Pb2       (II)      .       1.98 Info
PLAT909_ALERT_3_G Percentage of I>2sig(I) Data at Theta(Max) Still        68% Note
PLAT910_ALERT_3_G Missing # of FCF Reflection(s) Below Theta(Min).          4 Note
PLAT933_ALERT_2_G Number of HKL-OMIT Records in Embedded .res File          2 Note
PLAT941_ALERT_3_G Average HKL Measurement Multiplicity ..........         1.7 Low
PLAT978_ALERT_2_G Number C-C Bonds with Positive Residual Density.          0 Info
```

---

0 **ALERT level A** = Most likely a serious problem - resolve or explain
1 **ALERT level B** = A potentially serious problem, consider carefully
123 **ALERT level C** = Check. Ensure it is not caused by an omission or oversight
12 **ALERT level G** = General information/check it is not something unexpected

0 ALERT type 1 CIF construction/syntax error, inconsistent or missing data
15 ALERT type 2 Indicator that the structure model may be wrong or deficient
7 ALERT type 3 Indicator that the structure quality may be low
112 ALERT type 4 Improvement, methodology, query or suggestion
2 ALERT type 5 Informative message, check

It is advisable to attempt to resolve as many as possible of the alerts in all categories. Often the minor alerts point to easily fixed oversights, errors and omissions in your CIF or refinement strategy, so attention to these fine details can be worthwhile. In order to resolve some of the more serious problems it may be necessary to carry out additional measurements or structure refinements. However, the purpose of your study may justify the reported deviations and the more serious of these should normally be commented upon in the discussion or experimental section of a paper or in the "special_details" fields of the CIF. checkCIF was carefully designed to identify outliers and unusual parameters, but every test has its limitations and alerts that are not important in a particular case may appear. Conversely, the absence of alerts does not guarantee there are no aspects of the results needing attention. It is up to the individual to critically assess their own results and, if necessary, seek expert advice.

Publication of your CIF in IUCr journals

A basic structural check has been run on your CIF. These basic checks will be run on all CIFs submitted for publication in IUCr journals (*Acta Crystallographica*, *Journal of Applied Crystallography*, *Journal of Synchrotron Radiation*); however, if you intend to submit to *Acta Crystallographica Section C* or *E* or *IUCrData*, you should make sure that full publication checks are run on the final version of your CIF prior to submission.

Publication of your CIF in other journals

Please refer to the *Notes for Authors* of the relevant journal for any special instructions relating to CIF submission.

PLATON version of 06/07/2023; check.def file version of 30/06/2023

Datablock mo_22kub294_0m_a - ellipsoid plot

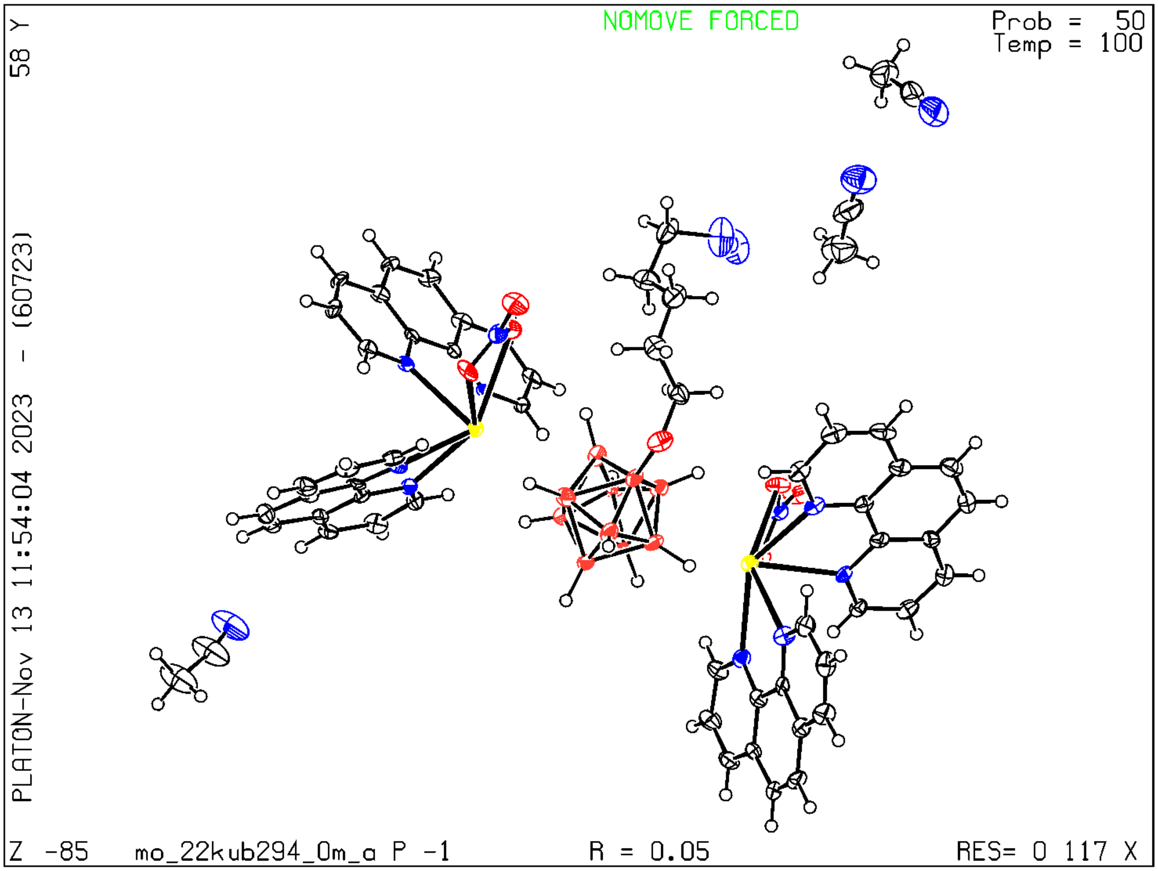

Supplement: Supplementary file 1 [file molecules-28-08073-s001.zip › 6.pdf]
