# Supplementary material for: Synthesis and Structures of Lead(II) Complexes with Substituted Derivatives of the Closo-Decaborate Anion with a Pendant N3 Group
Source: Molecules. 2023 Dec 13;28(24):8073. doi: 10.3390/molecules28248073 (PMC10746007; doi:10.3390/molecules28248073)

checkCIF/PLATON report

Structure factors have been supplied for datablock(s) mo_23kub21_0m_a

THIS REPORT IS FOR GUIDANCE ONLY. IF USED AS PART OF A REVIEW PROCEDURE FOR PUBLICATION, IT SHOULD NOT REPLACE THE EXPERTISE OF AN EXPERIENCED CRYSTALLOGRAPHIC REFEREE.

No syntax errors found. CIF dictionary Interpreting this report

Datablock: mo_23kub21_0m_a

```
Bond precision:   C-C = 0.0135 A           Wavelength=0.71073

Cell:             a=16.8622(8)     b=17.0582(8)       c=18.2240(9)
                  alpha=90         beta=109.153(2)    gamma=90
Temperature:      150 K
```

	Calculated	Reported
Volume	4951.8(4)	4951.8(4)
Space group	P 2/n	P 1 2/n 1
Hall group	-P 2yac	-P 2yac
Moiety formula	C28 H42 B20 N12 O10 Pb2, 2(C20 H16 N5 O3 Pb), C2 H3 N, 0.25(O)	C34 H37 B10 N11 O8 Pb2, 0.5(C2 H3 N), 0.125(O)
Sum formula	C70 H77 B20 N23 O16.25 Pb4	C35 H38.50 B10 N11.50 O8.12 Pb2
Mr	2545.55	1272.75
Dx,g cm-3	1.707	1.707
Z	2	4
Mu (mm-1)	6.849	6.849
F000	2432.0	2432.0
F000’	2406.51	
h,k,lmax		20,21,22
Nref		9718
Tmin,Tmax	0.568,0.934	0.473,0.745
Tmin’	0.252	

```
Correction method= # Reported T Limits: Tmin=0.473 Tmax=0.745
AbsCorr = MULTI-SCAN

Data completeness=                  Theta(max)= 26.045
```

```
R(reflections)= 0.0706( 6229)                                wR2(reflections)=
                                                             0.1868( 9718)
S = 1.014                 Npar= 273
```

The following ALERTS were generated. Each ALERT has the format
test-name_ALERT_alert-type_alert-level.
Click on the hyperlinks for more details of the test.

Alert level C

```
PLAT041_ALERT_1_C Calc. and Reported SumFormula    Strings  Differ     Please Check
              Calc: C35 H38.50 B10 N11.50 O8.13 Pb2
              Rep.: C35 H38.50 B10 N11.50 O8.12 Pb2
PLAT042_ALERT_1_C Calc. and Reported MoietyFormula Strings  Differ     Please Check
              Calc: C28 H42 B20 N12 O10 Pb2, 2(C20 H16 N5 O3 Pb), C2 H3 N, 0.25(
              Rep.: C34 H37 B10 N11 O8 Pb2, 0.5(C2 H3 N), 0.125(O)
PLAT077_ALERT_4_C Unitcell Contains Non-integer Number of Atoms ..     Please Check
PLAT218_ALERT_3_C Constrained U(ij) Components(s)  for C36        .          2 Check
PLAT218_ALERT_3_C Constrained U(ij) Components(s)  for C37        .          2 Check
PLAT222_ALERT_3_C NonSolvent Resd 1  H   Uiso(max)/Uiso(min) Range       10.0 Ratio
PLAT234_ALERT_4_C Large Hirshfeld Difference Pb2       --O3B      .       0.18 Ang.
PLAT234_ALERT_4_C Large Hirshfeld Difference O1        --C1       .       0.21 Ang.
PLAT241_ALERT_2_C High   'MainMol' Ueq as Compared to Neighbors of         C1 Check
PLAT242_ALERT_2_C Low    'MainMol' Ueq as Compared to Neighbors of        Pb2 Check
PLAT242_ALERT_2_C Low    'MainMol' Ueq as Compared to Neighbors of         O2 Check
PLAT242_ALERT_2_C Low    'MainMol' Ueq as Compared to Neighbors of        Pb1 Check
PLAT245_ALERT_2_C U(iso) H7A       Smaller than U(eq) B7        by      0.032 Ang**2
PLAT245_ALERT_2_C U(iso) H8A       Smaller than U(eq) B8        by      0.045 Ang**2
PLAT250_ALERT_2_C Large U3/U1 Ratio for Average U(i,j) Tensor ....        2.7 Note
PLAT260_ALERT_2_C Large Average Ueq of Residue Including       Pb2      0.109 Check
PLAT260_ALERT_2_C Large Average Ueq of Residue Including       N15      0.117 Check
PLAT342_ALERT_3_C Low Bond Precision on  C-C Bonds ..............    0.01354 Ang.
PLAT411_ALERT_2_C Short Inter H...H Contact  H6A      ..H28       .       2.08 Ang.
                                            1/2+x,2-y,1/2+z  =      4_676 Check
PLAT723_ALERT_1_C Torsion Calc       96.00, Rep      92(43) Dev...       4.00 Sigma
              C4A -N1A -N2A -N3A   1_555  1_555  1_555  1_555      #  105 Check
PLAT723_ALERT_1_C Torsion Calc       36.00, Rep      32(30) Dev...       4.00 Sigma
              C4B -N1B -N2B -N3B   1_555  1_555  1_555  1_555      #  107 Check
PLAT723_ALERT_1_C Torsion Calc      -14.00, Rep     -11(73) Dev...       3.00 Sigma
              C5A -N4A -N5A -N6A   1_555  1_555  1_555  1_555      #  109 Check
PLAT906_ALERT_3_C Large K Value in the Analysis of Variance ......      5.102 Check
PLAT911_ALERT_3_C Missing FCF Refl Between Thmin & STh/L=    0.600         55 Report
                2  0  0,   1  1  0,   2  2  0,  -1  1  1,  11 14  1,  -2  0  2,
              -10  7  4,  -9  8  4,  -7 10  4,  -6 11  4, -10  7  5,  -9  8  5,
                3 13  5,   7 17  5,  -7 18  5,  -8 17  6,  -5 19  6,  15  0  7,
              -17  8  7,  -7  9  7,  -9 16  7,   6  0  8,   8  0  8,  10  0  8,
               12  0  8,  14  0  8, -11 14  8,   9  0  9,  11  0  9,  13  0  9,
              -15 10  9, -14 11  9, -13 12  9,   4  3 10,   3  6 10,  -1  8 10,
              -10 14 10, -12 12 11,  -9 14 11,  -7 15 11,  11  3 12, -11 12 12,
               -1  0 13,   9  0 13, -13 10 13, -10 12 13,  -4  0 14,   7  7 14,
                6  9 14, -12 10 14,   3 12 14,   1 13 14,  -4 12 15, -12  8 16,
               -7  0 21,
PLAT972_ALERT_2_C Check Calcd Resid. Dens.  0.83Ang From Pb2            -1.68 eA-3
PLAT975_ALERT_2_C Check Calcd Resid. Dens.  0.76Ang From O9B      .       0.83 eA-3
PLAT977_ALERT_2_C Check Negative Difference Density on H5         .      -0.34 eA-3
```

```
PLAT977_ALERT_2_C Check Negative Difference Density on H14       .      -0.35 eA-3
PLAT977_ALERT_2_C Check Negative Difference Density on H23       .      -0.40 eA-3
PLAT977_ALERT_2_C Check Negative Difference Density on H27       .      -0.33 eA-3
```

Alert level G

```
PLAT002_ALERT_2_G Number of Distance or Angle Restraints on AtSite         31 Note
PLAT045_ALERT_1_G Calculated and Reported Z Differ by a Factor ...      0.500 Check
PLAT083_ALERT_2_G SHELXL Second Parameter in WGHT  Unusually Large      41.10 Why ?
PLAT171_ALERT_4_G The CIF-Embedded .res File Contains EADP Records         16 Report
PLAT172_ALERT_4_G The CIF-Embedded .res File Contains DFIX Records         40 Report
PLAT232_ALERT_2_G Hirshfeld Test Diff (M-X)  Pb2      --N9       .        8.5 s.u.
PLAT232_ALERT_2_G Hirshfeld Test Diff (M-X)  Pb2      --O3A      .        6.4 s.u.
PLAT232_ALERT_2_G Hirshfeld Test Diff (M-X)  Pb2      --O4A_a    .        7.0 s.u.
PLAT232_ALERT_2_G Hirshfeld Test Diff (M-X)  Pb1      --N11      .        6.0 s.u.
PLAT232_ALERT_2_G Hirshfeld Test Diff (M-X)  Pb1      --N12      .        7.0 s.u.
PLAT300_ALERT_4_G Atom Site Occupancy of O3B        Constrained at        0.6 Check
PLAT300_ALERT_4_G Atom Site Occupancy of O4B        Constrained at        0.6 Check
PLAT300_ALERT_4_G Atom Site Occupancy of O5A        Constrained at        0.8 Check
PLAT300_ALERT_4_G Atom Site Occupancy of O5B        Constrained at        0.6 Check
PLAT300_ALERT_4_G Atom Site Occupancy of N7B        Constrained at        0.6 Check
PLAT300_ALERT_4_G Atom Site Occupancy of O3A        Constrained at        0.4 Check
PLAT300_ALERT_4_G Atom Site Occupancy of O4A        Constrained at        0.4 Check
PLAT300_ALERT_4_G Atom Site Occupancy of C3B        Constrained at        0.6 Check
PLAT300_ALERT_4_G Atom Site Occupancy of N1A        Constrained at        0.2 Check
PLAT300_ALERT_4_G Atom Site Occupancy of N1B        Constrained at        0.3 Check
PLAT300_ALERT_4_G Atom Site Occupancy of N2A        Constrained at        0.2 Check
PLAT300_ALERT_4_G Atom Site Occupancy of N2B        Constrained at        0.3 Check
PLAT300_ALERT_4_G Atom Site Occupancy of N3A        Constrained at        0.2 Check
PLAT300_ALERT_4_G Atom Site Occupancy of N3B        Constrained at        0.3 Check
PLAT300_ALERT_4_G Atom Site Occupancy of N4A        Constrained at        0.2 Check
PLAT300_ALERT_4_G Atom Site Occupancy of N4B        Constrained at        0.3 Check
PLAT300_ALERT_4_G Atom Site Occupancy of N5A        Constrained at        0.2 Check
PLAT300_ALERT_4_G Atom Site Occupancy of N5B        Constrained at        0.3 Check
PLAT300_ALERT_4_G Atom Site Occupancy of N6A        Constrained at        0.2 Check
PLAT300_ALERT_4_G Atom Site Occupancy of N6B        Constrained at        0.3 Check
PLAT300_ALERT_4_G Atom Site Occupancy of N7A        Constrained at        0.4 Check
PLAT300_ALERT_4_G Atom Site Occupancy of C3A        Constrained at        0.4 Check
PLAT300_ALERT_4_G Atom Site Occupancy of C4A        Constrained at        0.2 Check
PLAT300_ALERT_4_G Atom Site Occupancy of C4B        Constrained at        0.3 Check
PLAT300_ALERT_4_G Atom Site Occupancy of C5A        Constrained at        0.2 Check
PLAT300_ALERT_4_G Atom Site Occupancy of C5B        Constrained at        0.3 Check
PLAT300_ALERT_4_G Atom Site Occupancy of O6A        Constrained at        0.5 Check
PLAT300_ALERT_4_G Atom Site Occupancy of O6B        Constrained at        0.5 Check
PLAT300_ALERT_4_G Atom Site Occupancy of O7A        Constrained at        0.5 Check
PLAT300_ALERT_4_G Atom Site Occupancy of O7B        Constrained at        0.5 Check
PLAT300_ALERT_4_G Atom Site Occupancy of O8A        Constrained at        0.5 Check
PLAT300_ALERT_4_G Atom Site Occupancy of O8B        Constrained at        0.5 Check
PLAT300_ALERT_4_G Atom Site Occupancy of N10A       Constrained at        0.5 Check
PLAT300_ALERT_4_G Atom Site Occupancy of N10B       Constrained at        0.5 Check
PLAT300_ALERT_4_G Atom Site Occupancy of C36        Constrained at        0.5 Check
PLAT300_ALERT_4_G Atom Site Occupancy of C37        Constrained at        0.5 Check
PLAT300_ALERT_4_G Atom Site Occupancy of H37A       Constrained at        0.5 Check
PLAT300_ALERT_4_G Atom Site Occupancy of H37B       Constrained at        0.5 Check
PLAT300_ALERT_4_G Atom Site Occupancy of H37C       Constrained at        0.5 Check
PLAT300_ALERT_4_G Atom Site Occupancy of O9B        Constrained at       0.25 Check
PLAT301_ALERT_3_G Main Residue  Disorder .............(Resd  1  )         26% Note
```

```
PLAT302_ALERT_4_G Anion/Solvent/Minor-Residue Disorder (Resd  2  )        14% Note
PLAT302_ALERT_4_G Anion/Solvent/Minor-Residue Disorder (Resd  3  )        67% Note
PLAT302_ALERT_4_G Anion/Solvent/Minor-Residue Disorder (Resd  4  )       100% Note
PLAT303_ALERT_2_G Full Occupancy Atom H9A       with # Connections       2.00 Check
PLAT304_ALERT_4_G Non-Integer Number of Atoms in ..... (Resd  4  )       0.12 Check
PLAT311_ALERT_2_G Isolated Disordered Oxygen Atom (No H's ?) .....        O9B Check
PLAT432_ALERT_2_G Short Inter X...Y Contact  C23      ..C5A      .       3.09 Ang.
                                                    x,1+y,z  =      1_565 Check
PLAT789_ALERT_4_G Atoms with Negative _atom_site_disorder_group  #         23 Check
PLAT790_ALERT_4_G Centre of Gravity not Within Unit Cell: Resd.  #          4 Note
              O
PLAT811_ALERT_5_G No ADDSYM Analysis: Too Many Excluded Atoms ....          ! Info
PLAT822_ALERT_4_G CIF-embedded .res Contains Negative PART Numbers         19 Check
PLAT860_ALERT_3_G Number of Least-Squares Restraints ............          38 Note
PLAT883_ALERT_1_G No Info/Value for _atom_sites_solution_primary .     Please Do !
PLAT910_ALERT_3_G Missing # of FCF Reflection(s) Below Theta(Min).          2 Note
                0  1  0,  -1  0  1,
PLAT912_ALERT_4_G Missing # of FCF Reflections Above STh/L=  0.600         14 Note
PLAT933_ALERT_2_G Number of HKL-OMIT Records in Embedded .res File          3 Note
               -1  1  1,   2  0  0,   2  2  0,
PLAT941_ALERT_3_G Average HKL Measurement Multiplicity ..........         2.0 Low
PLAT978_ALERT_2_G Number C-C Bonds with Positive Residual Density.          0 Info
```

---

   0 **ALERT level A** = Most likely a serious problem - resolve or explain
   0 **ALERT level B** = A potentially serious problem, consider carefully
  30 **ALERT level C** = Check. Ensure it is not caused by an omission or oversight
  69 **ALERT level G** = General information/check it is not something unexpected

   7 ALERT type 1 CIF construction/syntax error, inconsistent or missing data
  28 ALERT type 2 Indicator that the structure model may be wrong or deficient
  10 ALERT type 3 Indicator that the structure quality may be low
  53 ALERT type 4 Improvement, methodology, query or suggestion
   1 ALERT type 5 Informative message, check

It is advisable to attempt to resolve as many as possible of the alerts in all categories. Often the minor alerts point to easily fixed oversights, errors and omissions in your CIF or refinement strategy, so attention to these fine details can be worthwhile. In order to resolve some of the more serious problems it may be necessary to carry out additional measurements or structure refinements. However, the purpose of your study may justify the reported deviations and the more serious of these should normally be commented upon in the discussion or experimental section of a paper or in the "special_details" fields of the CIF. checkCIF was carefully designed to identify outliers and unusual parameters, but every test has its limitations and alerts that are not important in a particular case may appear. Conversely, the absence of alerts does not guarantee there are no aspects of the results needing attention. It is up to the individual to critically assess their own results and, if necessary, seek expert advice.

Publication of your CIF in IUCr journals

A basic structural check has been run on your CIF. These basic checks will be run on all CIFs submitted for publication in IUCr journals (*Acta Crystallographica*, *Journal of Applied Crystallography*, *Journal of Synchrotron Radiation*); however, if you intend to submit to *Acta Crystallographica Section C* or *E* or *IUCrData*, you should make sure that full publication checks are run on the final version of your CIF prior to submission.

Publication of your CIF in other journals

Please refer to the *Notes for Authors* of the relevant journal for any special instructions relating to CIF submission.

PLATON version of 29/11/2023; check.def file version of 14/09/2023

Datablock mo_23kub21_0m_a - ellipsoid plot

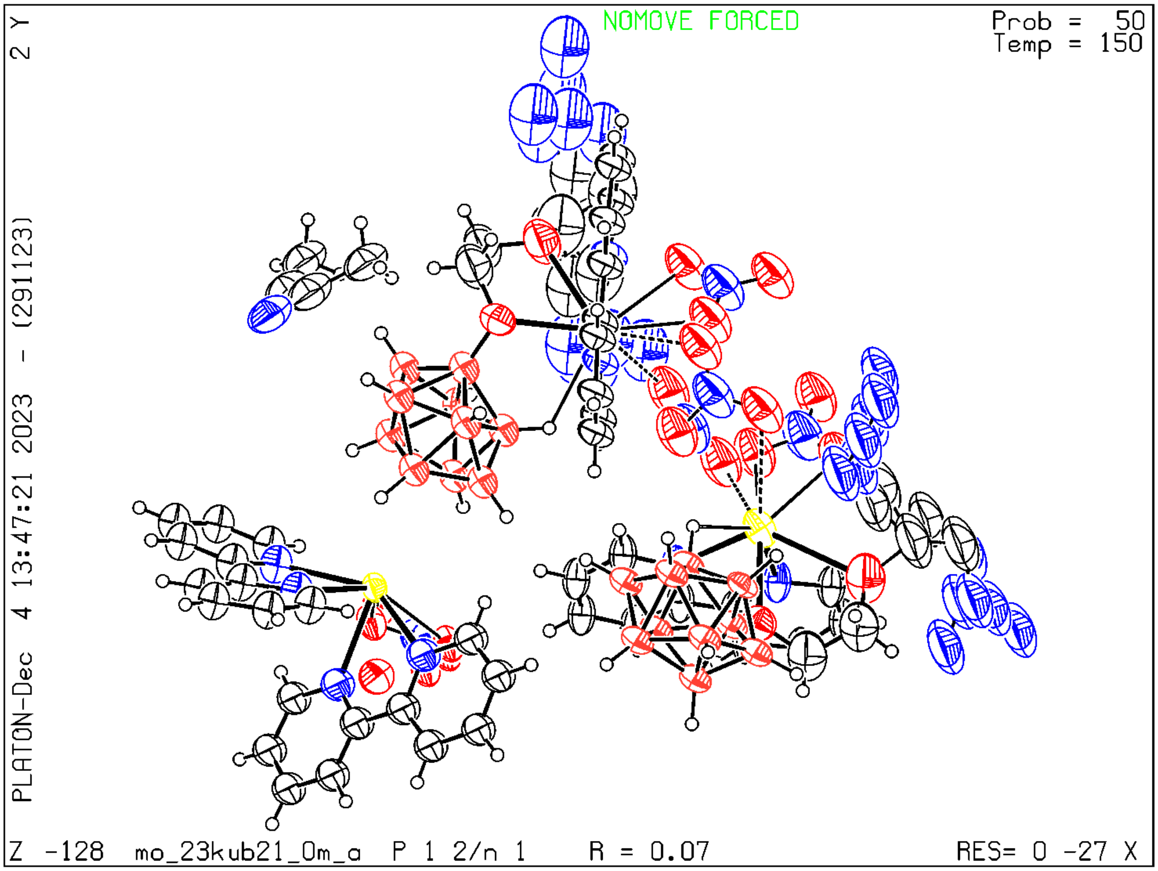

Supplement: Supplementary file 1 [file molecules-28-08073-s001.zip › 7.pdf]
