# Supplementary material for: Synthesis and Structures of Lead(II) Complexes with Substituted Derivatives of the Closo-Decaborate Anion with a Pendant N3 Group
Source: Molecules. 2023 Dec 13;28(24):8073. doi: 10.3390/molecules28248073 (PMC10746007; doi:10.3390/molecules28248073)
Supplement: Supplementary file 1 [file molecules-28-08073-s001.zip › SI.pdf]

Table S1. Crystal data and structure refinement for compounds 2–7.

**Compound**	**2**	**4**	**6·3CH3CN**	**7· 0.5CH3CN·0.125H2O***
Empirical formula	$C_{53}H_{59}B_{10}N_{3}OP_{2}$	$C_{28}H_{33}B_{10}N_{7}O_{2}Pb$	$C_{59}H_{60}B_{10}N_{16}O_{7}Pb_{2}$	$C_{35}H_{38.5}B_{10}N_{11.5}O_{8.13}Pb_{2}$
Formula weight	924.07	814.90	1627.71	1272.75
Temperature, K	100.00	150.00	100.00	150.00
Crystal system	monoclinic	triclinic	triclinic	monoclinic
Space group	$P2_1/c$	P-1	P-1	P2/n
a, Å	19.758(6)	11.273(5)	9.377(3)	16.8622(8)
b, Å	13.276(2)	13.638(8)	17.981(6)	17.0582(8)
c, Å	19.385(7)	22.354(14)	19.298(10)	18.2240(9)
α, °	90	73.72(2)	99.786(19)	90
β, °	96.157(14)	87.76(2)	95.463(18)	109.153(2)
γ, °	90	88.16(2)	97.987(9)	90
Volume, $Å^3$	5056(3)	3296(3)	3152(2)	4951.8(4)
Z	4	4	2	4
ρ_{calc}g, cm^3	1.214	1.642	1.715	1.707
μ, mm^{-1}	0.128	5.160	5.402	6.849
F(000)	1944.0	1592.0	1592.0	2432.0
2Θ range, °	3.702 to 55.796	3.16 to 61.06	4.316 to 50.206	3.362 to 52.09
Index ranges	-16 ≤ h ≤ 26, -17 ≤ k ≤ 16, -21 ≤ l ≤ 25	-15 ≤ h ≤ 15, -19 ≤ k ≤ 19, -30 ≤ l ≤ 31	-11 ≤ h ≤ 11, -21 ≤ k ≤ 21, -22 ≤ l ≤ 23	-19 ≤ h ≤ 20, -21 ≤ k ≤ 18, -22 ≤ l ≤ 14
Reflections collected	21776	30205	18658	19673
Independent reflections	11803 [R_{int} = 0.0740, R_{sigma} = 0.1426]	18856 [R_{int} = 0.0872, R_{sigma} = 0.1642]	10974 [R_{int} = 0.0361, R_{sigma} = 0.0689]	9718 [R_{int} = 0.0423, R_{sigma} = 0.0811]
Goodness-of-fit on F^2	1.036	1.025	1.074	1.014
Final R indexes [I>=2σ (I)]	R_1 = 0.0789, wR_2 = 0.1733	R_1 = 0.0797, wR_2 = 0.1833	R_1 = 0.0517, wR_2 = 0.1057	R_1 = 0.0706, wR_2 = 0.1628
Final R indexes [all data]	R_1 = 0.1589, wR_2 = 0.2253	R_1 = 0.1350, wR_2 = 0.2117	R_1 = 0.0687, wR_2 = 0.1127	R_1 = 0.1173, wR_2 = 0.1868

*Structure **7**· $0.5CH_3CN·0.125H_2O$ contains an isolated O atom in the unit cell with the 0.125 occupancy, that was classified as a water molecule.
